# Fast A‐Site Cation Cross‐Exchange at Room Temperature: Single‐to Double‐ and Triple‐Cation Halide Perovskite Nanocrystals

**DOI:** 10.1002/anie.202205617

**Published:** 2022-07-13

**Authors:** Clara Otero‐Martínez, Muhammad Imran, Nadine J. Schrenker, Junzhi Ye, Kangyu Ji, Akshay Rao, Samuel D. Stranks, Robert L. Z. Hoye, Sara Bals, Liberato Manna, Jorge Pérez‐Juste, Lakshminarayana Polavarapu

**Affiliations:** ^1^ Department of Physical Chemistry, CINBIO Universidade de Vigo, Materials Chemistry and Physics Group Campus Universitario As Lagoas, Marcosende 36310 Vigo Spain; ^2^ Department of Physical Chemistry, CINBIO Universidade de Vigo Campus Universitario As Lagoas, Marcosende 36310 Vigo Spain; ^3^ Nanochemistry Istituto Italiano di Tecnologia Via Morego 30 16163 Genova Italy; ^4^ EMAT and Nanolab Center of Excellence University of Antwerp Groenenborgerlaan 171 2020 Antwerp Belgium; ^5^ Cavendish Laboratory University of Cambridge 19 JJ Thomson Avenue Cambridge CB3 0HE UK; ^6^ Department of Chemical Engineering and Biotechnology University of Cambridge Cambridge CB3 0AS UK; ^7^ Department of Materials Imperial College London Exhibition Road London SW7 2AZ UK

**Keywords:** Cation Exchange, Mixed A-Cation Perovskites, Perovskite Nanocrystals, Phase Segregation, Triple-Cation Perovskites

## Abstract

We report here fast A‐site cation cross‐exchange between APbX_3_ perovskite nanocrystals (NCs) made of different A‐cations (Cs (cesium), FA (formamidinium), and MA (methylammonium)) at room temperature. Surprisingly, the A‐cation cross‐exchange proceeds as fast as the halide (X=Cl, Br, or I) exchange with the help of free A‐oleate complexes present in the freshly prepared colloidal perovskite NC solutions. This enabled the preparation of double (MACs, MAFA, CsFA)‐ and triple (MACsFA)‐cation perovskite NCs with an optical band gap that is finely tunable by their A‐site composition. The optical spectroscopy together with structural analysis using XRD and atomically resolved high‐angle annular dark‐field scanning transmission electron microscopy (HAADF‐STEM) and integrated differential phase contrast (iDPC) STEM indicates the homogeneous distribution of different cations in the mixed perovskite NC lattice. Unlike halide ions, the A‐cations do not phase‐segregate under light illumination.

## Graphical abstract

The A‐cation cross‐exchange between perovskite nanocrystals (NCs) is found to be as fast as the halide exchange in the presence of excess A‐oleate complexes in the colloidal solution. The synthesis of a wide range of mixed (double and triple)‐cation and mixed halide perovskite NCs is demonstrated. The mixed A‐cations were found to be uniformly distributed across the crystal lattice and they do not segregate under intense light illumination.

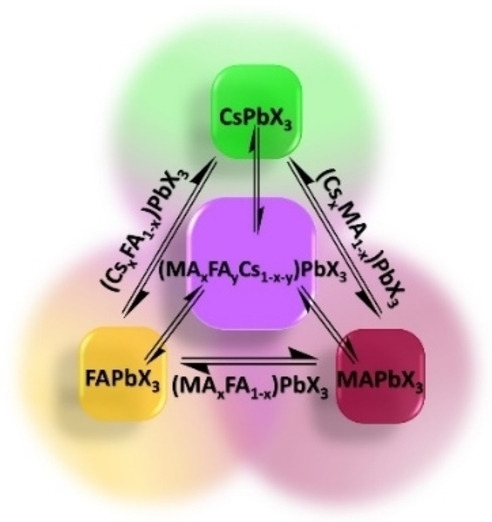

## Introduction

In recent years, lead halide perovskite nanocrystals (LHP NCs) have been receiving significant attention owing to their interesting properties and potential applications.^[1]^ In particular, the chemistry of LHP NCs has been greatly exploited to tune their optical and electronic properties as well as for improving their stability.[^1a^, ^2^] LHP NCs exhibit near‐unity photoluminescence quantum yields (PLQYs) under optimal growth conditions and their emission color is finely tunable across the visible spectrum of light by their composition (APbX_3_; A=methylammonium (MA), cesium (Cs), formamidinium (FA); X=Cl, Br, I).[^1a^, ^1b^, ^3^] The composition of LHP NCs is tunable either by direct synthesis or through post‐synthetic ion exchange.[^1b^, ^3d^, ^3e^, ^4^] In general, ion exchange reactions provide access to NCs with precise chemical compositions that may be difficult to access by direct synthetic routes.[^1a^, ^1b^, ^3e^, ^5^] This strategy has been widely used to tune the halide composition and thus optical band gap of in LHP NCs.[^1a^, ^1b^, ^3e^, ^5a^] Halide exchange in LHPs takes place spontaneously at room temperature like cation exchange in chalcogenides.^[6]^ However, its analogous A‐site cation exchange in LHPs remains relatively unexplored to date. Unlike halide exchange, cation exchange in perovskites has a high activation energy^[7]^ and the exchange process can distort or dismantle the lattice structures due to their large size.

On the other hand, mixed cation perovskite thin films have been found to be promising candidates for solar cells with improved thermal and structural stability, as well as reproducible power conversion efficiency.^[8]^ The improved structural stability has been attributed to the tuning of the Goldschmidt tolerance factor by varying the ratio of Cs/FA/MA ions.^[8a]^ The mixed cation perovskite films are often prepared by direct synthesis because it is difficult to perform post‐synthetic ion exchange in films.^[9]^ In fact, direct synthesis of the mixed cation perovskite films with controlled composition is also challenging due to significant differences in the crystallization temperatures of the corresponding mono‐cation perovskite films.[^8a^, ^10^] Recently, a few studies have shown the formation of A‐site double‐cation (Cs/FA) colloidal LHP NCs with controlled composition by post‐synthetic cation cross‐exchange.[^7^, ^11^] For instance, Luther's group demonstrated the A‐site cation cross‐exchange between LHP NCs made of two different cations (Cs and FA).[^7^, ^11b^] However, unlike halide cross‐exchange,[^1b^, ^3e^] A‐site cation cross‐exchange did not occur spontaneously.^[7]^ Instead, it took around 24 hours to complete the A‐site cation cross‐exchange at 45 °C.^[7]^ Recently, Hao et al.^[11c]^ reported that the cation cross‐exchange completes in 30 min in the presence of oleic acid ligands. The resultant Cs_*x*_FA_1−*x*_PbI_3_ NCs were found to have small domains of pure CsPbI_3_ and FAPbI_3_ in the crystal lattice.^[11c]^ Despite these few studies, A‐site cation exchange has not been fully exploited towards the compositional tunability of perovskite NCs, e.g. triple cation NCs. Moreover, the mechanism of A‐site cation cross‐exchange is rather complicated as the NC surface ligand plays a crucial role and it needs in‐depth understanding.

**Figure 1:**
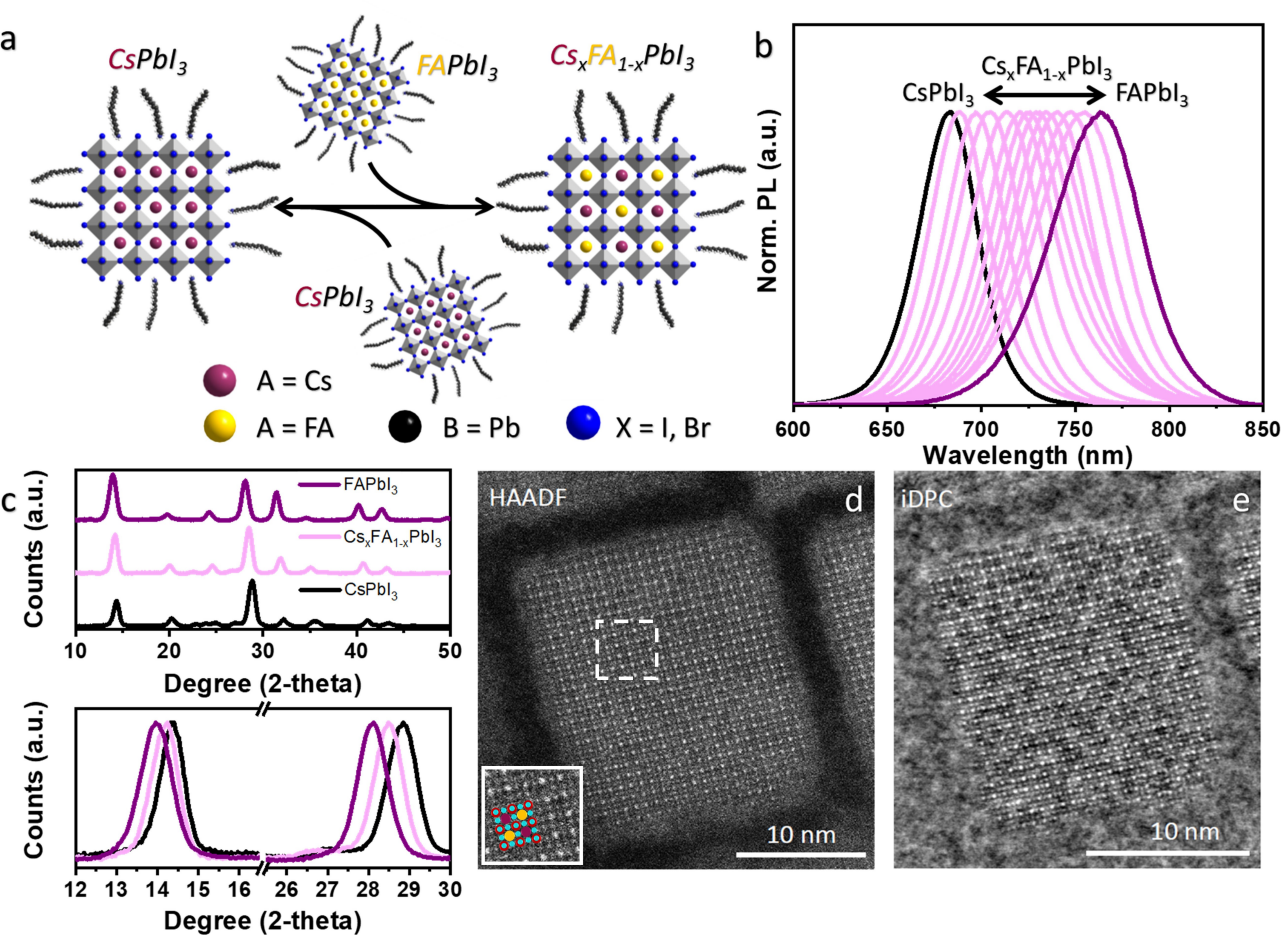
a) Schematic illustration of the formation of double‐cation perovskite (Cs_*x*_FA_1−*x*_PbI_3_) NCs through reversible cation exchange by mixing CsPbI_3_ and FAPbI_3_ perovskite NCs. b) Normalized PL spectra of CsPbI_3_, FAPbI_3_ and Cs_*x*_FA_1−*x*_PbI_3_ NCs obtained by cation exchange. The emission wavelength of the mixed‐cation NCs is tunable between those of the individual pure A‐cation compositions. c) XRD diffraction pattern of CsPbI_3_, FAPbI_3_ and Cs_*x*_FA_1−*x*_PbI_3_ NCs obtained by cation exchange. In the bottom panel, the enlarged spectra evidence the peak shift that confirms the mixed cation composition. d,e) Representative high resolution HAADF‐STEM (d) and integrated differential phase contrast (iDPC)‐STEM (e) images of Cs_1−*x*_FA_*x*_PbI_3_ NCs (PL peak at 725 nm). The uniform intensity in the HAADF‐STEM image indicates that there is no segregation of the two cations in the lattice. Due to the mix of Cs and FA, the intensity of the A‐site atomic columns is very low and hardly detectable in the HAADF image. However, the phase contrast in the iDPC image enables visualization of the Cs atoms. The overlay in the inset of panel (d) indicates the atom column position of Cs (purple), FA (yellow), Pb (red) and the halide (blue) in the perovskite structure. The Cs and FA cations are randomly mixed.

Herein, we report the spontaneous A‐site cation (MA, FA, and Cs) cross‐exchange at room temperature in a mixture of freshly prepared halide perovskite NCs made of different A‐site cations (Figure 1a). This cross‐exchange is found to be as fast as the typical halide (Cl, Br, and I) exchanges. We thus demonstrate the preparation of double and triple cation perovskite NCs with variable A‐site cation and halide combinations and compositions. The cross‐exchange performed under different ligand environments revealed that the excess A‐oleates (for example, Cs‐oleate and FA‐oleate) present in the freshly prepared unpurified NCs trigger the cation cross‐exchange and that the removal of these oleates by purification using an antisolvent significantly slows down the cross‐exchange process. Atomically‐resolved HAADF‐STEM analysis with a low electron dose suggests a homogeneous distribution of A‐site cations across the perovskite crystal lattice.

## Results and Discussion

To study the A‐site cation cross‐exchange, colloidal CsPbI_3_ and FAPbI_3_ perovskite NCs are first synthesized by a hot‐plate approach under ambient conditions and are dispersed in toluene (see the Supporting Information for details on synthesis and characterization of the NCs).^[3b]^ The colloidal dispersion of CsPbI_3_ and FAPbI_3_ perovskite NCs exhibited photoluminescence (PL) peaks at ≈680 and 770 nm, respectively (Figure S1, S2). Figure 1a outlines the reversible cation cross‐between freshly prepared FAPbI_3_ and CsPbI_3_ NCs to obtain Cs_*x*_FA_1−*x*_PbI_3_ NCs, and the process can be easily probed through PL spectroscopy. First, different volumes of the as‐synthesized colloidal dispersion of FAPbI_3_ NC were added to a freshly prepared CsPbI_3_ NC dispersion at room temperature to obtain Cs_*x*_FA_1−*x*_PbI_3_ NCs with different Cs/FA ratios (similar results were obtained by adding different volumes of CsPbI_3_ to FAPbI_3_). Figure 1b shows the normalized PL spectra of the resultant Cs_*x*_FA_1−*x*_PbI_3_ colloidal dispersions in comparison with those of pure CsPbI_3_ and FAPbI_3_ NCs. The reaction appeared to occur spontaneously after mixing the two colloidal dispersions. The mixed colloidal dispersions exhibited a single PL peak that red‐shifts toward the lower energy side by increasing the amount of added FAPbI_3_ NC solution. These results clearly suggest the formation of Cs_*x*_FA_1−*x*_PbI_3_ NCs with homogeneous alloy (Cs/FA) composition through A‐site cation exchange. The PL peak position of the Cs_*x*_FA_1−*x*_PbI_3_ NCs was precisely tunable between 680 and 775 nm by varying the ratio of the pure components that are mixed together. Furthermore, the cation exchange was reversible, meaning that the mixed A‐site cation perovskite NCs could be further converted into their pure counterparts by an excess addition of the respective pristine NC suspensions. It is important to note that the reaction speed lowers for aged NCs, likely due to the reduction in the concentration of free A‐oleate molecules over time. However, it is still faster compared to the reaction in NCs obtained by purification using antisolvents.

The bright‐field transmission electron microscope (TEM) images revealed that the Cs_*x*_FA_1−*x*_PbI_3_ NCs retain the original cubic shape (Figure S1–S3 for TEM images of CsPbI_3_, FAPbI_3,_ and Cs_*x*_FA_1−*x*_PbI_3_ NCs). Importantly, the Cs_*x*_FA_1−*x*_PbI_3_ NCs exhibited bimodal size distribution because of the two distinct size distributions of the pristine CsPbI_3_ (size ≈11.1+1.4 nm) and FAPbI_3_ (size ≈14.2+2.3 nm) NCs (Figure S4). Interestingly, this distribution did not have a significant influence on the PL peak position of the Cs_*x*_FA_1−*x*_PbI_3_ NCs and they still exhibited a single PL peak. This observation is rationalized by considering that the mean size for both size distributions is larger than the exciton Bohr diameter (Figure 1b). The structural characterization using powder X‐ray diffraction (XRD) of the starting samples (CsPbI_3_ and FAPbI_3_) and mixed cations NCs sample (prepared by mixing CsPbI_3_ and FAPbI_3_) is reported in Figure 1c. The XRD patterns of the starting CsPbI_3_ NCs unambiguously conformed to the “black” orthorhombic perovskite phase (of CsPbI_3_), whereas the XRD pattern of FAPbI_3_ matches closely with cubic bulk perovskite phase in agreement with previous reports. On the other hand, in the case of mixed cation sample (Cs_*x*_FA_1−*x*_PbI_3_ NCs with PL peak centered at 725 nm), the XRD peaks is centered in between their pure counterparts (see the zoomed portion of the main diffraction peaks in the bottom panel of Figure 1c). We ascribe such structural uniformity to the rapid alloying of the A‐sites upon mixing and suggest a homogeneous distribution of the two cations in the perovskite crystal lattice.

To gain further insight into the structural features and in particular the distribution of Cs and FA cations in the crystal lattice, we performed high‐angle annular dark‐field scanning transmission electron microscopy (HAADF‐STEM) of individual NCs. The Cs_*x*_FA_1−*x*_PbI_3_ NCs having PL peak at 725 nm were investigated as a model system for HAADF‐STEM analysis of mixed‐cation perovskite NCs. Considering the well‐known beam sensitivity of halide perovskites, the HAADF‐STEM images presented here were acquired using a low electron dose to avoid degradation under the electron beam. From atomically resolved HAADF‐STEM images of the Cs_*x*_FA_1−*x*_PbI_3_ NCs acquired along the [100] zone axis (Figure 1d) an average lattice parameter of 6.40±0.008 Å was determined, where the error corresponds to the standard error on the mean value (see Figure S5 for lattice spacing analysis, which was performed using the StatSTEM software).^[12]^ The A‐site columns in the HAADF‐STEM image of Cs_*x*_FA_1−*x*_PbI_3_ NCs exhibited higher contrast than those in FAPbI_3_ NCs due to the higher Z‐contrast of Cs compared to FA (see Figure S5 for the pure FAPbI_3_ NCs, the initial perovskite structure could be imaged without the formation of the degradation product PbI_2_).[^1a^, ^4^, ^13^] Due to the low intensity of the A‐site columns in HAADF images of mixed‐cation Cs_*x*_FA_1−*x*_PbI_3_ NCs, we further employed integrated differential phase contrast (iDPC) STEM imaging to study the distribution of Cs atoms in the crystal lattice (Figure 1e). The iDPC‐STEM image correlates to the scalar electrostatic potential field of the sample and is therefore a direct phase image for thin samples.^[14]^ Thus, it allows simultaneous imaging of both light and heavier elements. The homogeneous intensity for the different A‐site columns in the iDPC‐STEM image of the Cs_*x*_FA_1−*x*_PbI_3_ NCs and the lattice parameter analysis indicate that the Cs and FA cations were uniformly distributed across the crystal (Figure 1e). This is in contrast to previous observations of mixed‐cation perovskite NCs, in which the cation cross‐exchange led to the formation of small FAPbI_3_ domains, which transformed already to PbI_2_, and CsPbI_3_ domains distributed in the three‐dimensional crystal lattice.^[11c]^ In our case, the spontaneous cation exchange instead appears to lead to a uniform distribution of the two cations in the NCs. Furthermore, a single PL peak (narrower compared to pure FAPbI_3_ sample), intermediate between those of the pure materials, is also a strong argument in favour of homogeneous cation mixing in the NC lattice. (Figure 1b). Indeed, if there was phase segregation of Cs and FA ions, either the PL spectra would contain multiple PL peaks or a single and broad peak would originate from the FAPbI_3_ domains only, regardless of the cation composition, due to energy transfer from Cs domains to FA domains.^[15]^ Therefore, based on all these results, we conclude that there is no phase segregation of Cs and FA cations in the Cs_*x*_FA_1−*x*_PbI_3_ NCs obtained by spontaneous A‐site cation exchange. One of the most intriguing features in the formation of the Cs_*x*_FA_1−*x*_PbI_3_ NCs in this work is the speed at which the A‐site cation cross‐exchange occurs between CsPbI_3_ and FAPbI_3_ colloidal NCs upon mixing them at room temperature.

**Figure 2:**
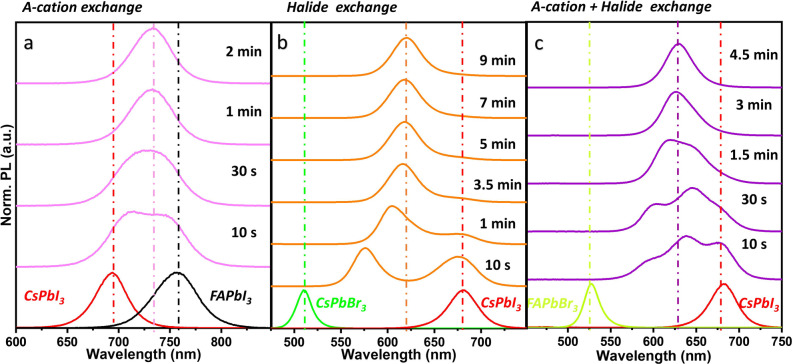
Kinetics of the cross exchange of A‐site cation (a), halide ion (b), and both (c) at room temperature, probed by time‐dependent PL emission. The evolution of the PL spectra of the colloidal solutions obtained after mixing the respective NCs in each case with respect to their individual PL spectra. The PL spectra of the corresponding individual NCs are presented at the bottom in all the figures. The PL spectra were acquired until the two individual emission peaks merged into single one and there was no further shift in it in all the three reactions.

To probe the cross‐exchange of Cs and FA cations more accurately, we acquired the time‐dependent PL of the resultant colloidal solution immediately after mixing the freshly prepared CsPbI_3_ and FAPbI_3_ NCs at room temperature (Figure 2a, see Supporting Information for experimental details). In the time scale of a few seconds after mixing, the PL peak of CsPbI_3_ started to red shift while the PL peak of FAPbI_3_ NCs blue shifted, suggesting the cross‐exchange of Cs and FA cations in the colloidal solution (Figure 2a). The two peaks eventually merged into a single one within 1–2 minutes, indicating the rapid completion of cation cross‐exchange to obtain a homogeneous colloidal solution Cs_*x*_FA_1−*x*_PbI_3_ NCs (Figure 2a). Importantly, the rapidity of the A‐site cation cross‐exchange is reminiscent of the halide ion (Br and I) cross‐exchange, which usually takes place also in a few minutes (this process as well can be tracked by monitoring the PL of the colloidal solution after mixing CsPbBr_3_ and CsPbI_3_ NCs).[^1b^, ^3e^] As depicted in Figure 2b, the emission peaks of CsPbBr_3_ and CsPbI_3_ NCs gradually merge into a single PL peak over the time of ≈7–9 min, which is consistent with the time range reported in previous studies.[^1b^, ^3e^] It is worth mentioning that the halide cross‐exchange takes much longer than direct exchange using a halide precursor (≈few minutes vs. few seconds).^[1b]^ Nevertheless, these results clearly suggest that the A‐site cation cross‐exchange takes place as fast as the halide ion cross‐exchange (Figures 2a, b). Furthermore, the simultaneous cross‐exchange of both A‐site cations and halide ions in a mixture of FAPbBr_3_ and CsPbI_3_ confirms that the cation cross‐exchange takes place as fast as the halide cross‐exchange, as illustrated in Figure 2c.

**Figure 3:**
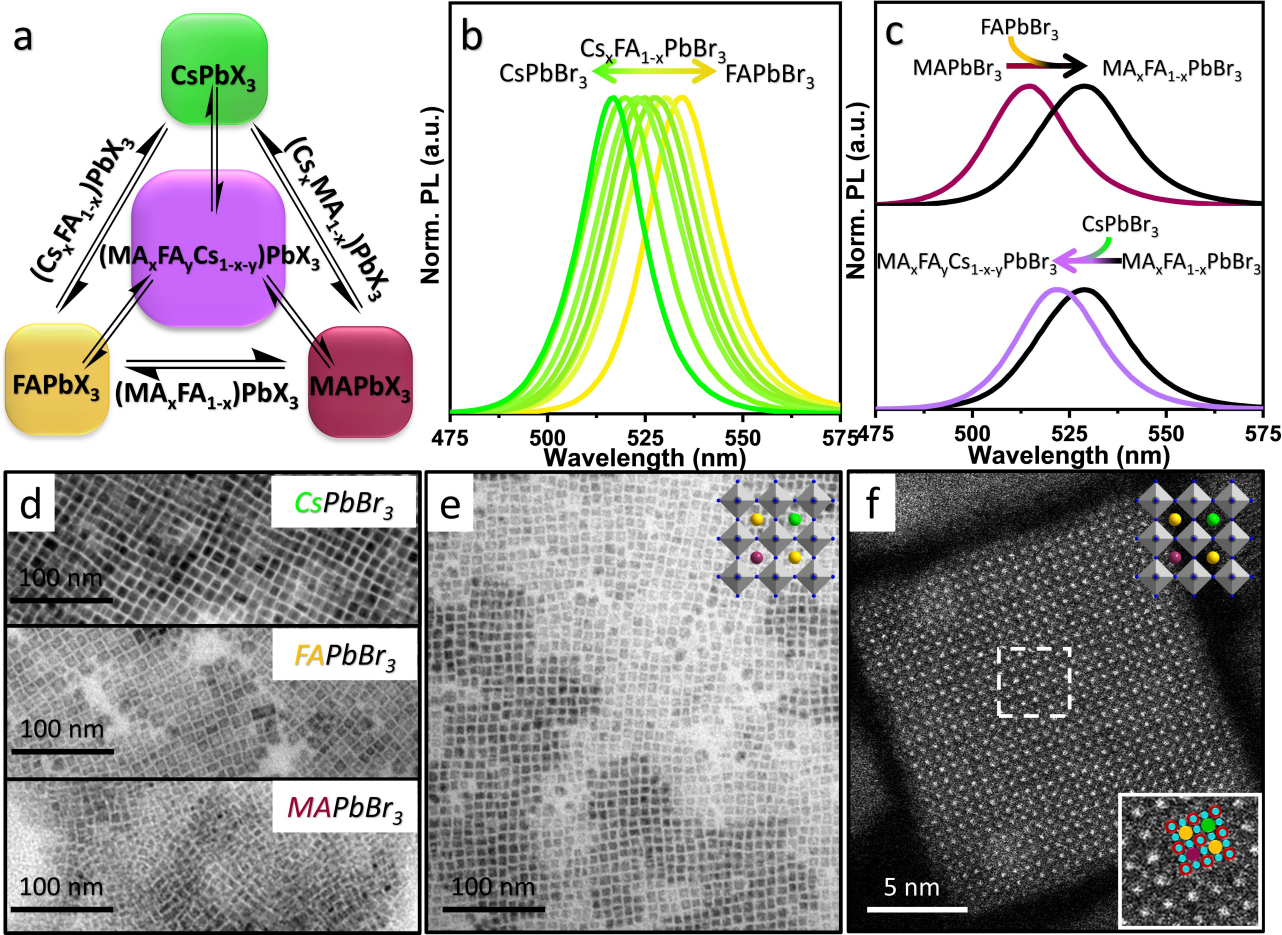
a) Schematic illustration showing different combinations of double (MAFA, CsMA, and CsFA) and triple (MAFACs)‐cation perovskite NCs that can be achieved through cation exchange by mixing the respective mono (MA, FA and Cs)‐cation perovskite NCs. b) Normalized PL spectra of colloidal Cs_*x*_FA_1−*x*_PbBr_3_ NCs obtained by mixing CsPbBr_3_ and FAPbBr_3_ NCs in different ratios. The emission wavelength of the mixed cation perovskite NCs is tunable in between their individual counterparts. c) Probing the formation of triple‐cation perovskite (MA_*x*_FAyCs_1−*x*−*y*_PbBr_3_) NCs by their photoluminescence. First, adding a colloidal dispersion of FAPbBr_3_ NCs into MAPbBr_3_ NC dispersion leads to a red shift of PL, suggesting the formation of MA_*x*_FA_1−*x*_PbBr_3_ NCs. Second, the addition of CsPbBr_3_ NCs to MA_*x*_FA_1−*x*_PbBr_3_ NCs results in a blue shift, indicating the cation exchange in between the two types of NCs, leading to the formation of MA_*x*_FAyCs_1−*x*−*y*_PbBr_3_. d) TEM images of CsPbBr_3_, FAPbBr_3_ and MAPbBr_3_ NCs (top to bottom) d,e) TEM (e) and high resolution HAADF‐STEM image (f) of the corresponding triple cation perovskite NCs prepared by mixing MAPbBr3, CsPbBr3 and FAPbBr3 NCs. The top insets of e and f are the schematic illustration of the crystal structures of the triple cation NCs. The overlay in the inset of panel (f) indicates the atom column position of Cs (green), FA (yellow), MA (purple), Pb (red) and the halide (blue) in the perovskite structure. The Cs, FA and MA cations are randomly mixed.

In principle, the fast cross‐exchange of A‐site cation and halide ion enables the preparation of perovskite NCs in a wide range of mixed (double and triple)‐cation and halide combinations by mixing the corresponding individual NCs, as illustrated in Figure 3a. First, we extended the A‐site cation exchange to Br‐based perovskite NCs (CsPbBr_3_ and FAPbBr_3_), and the reaction was probed by PL emission. The pristine CsPbBr_3_ and FAPbBr_3_ NCs exhibited PL emission peaks at 516 nm and 534 nm, respectively. Upon mixing them, the cation exchange took place in a matter of seconds and the two PL peaks merged into a single one, suggesting the formation of double‐cation perovskite NCs. As shown in Figure 3b, their emission wavelength was reversibly tunable in between 516 nm and 534 nm by mixing the two colloidal solutions in different ratios. It should be noted that the probing of cation exchange between CsPbBr_3_ NCs and MAPbBr_3_ NCs by PL emission is difficult because of the overlap of their emission spectra. Time‐resolved PL measurements revealed that the mixed cation NCs are in‐between their pure counterparts and the decay is highly dependent on their A‐cation composition (Figure S6 and Table S1). We further extended the cross‐exchange to obtain triple (MACsFA)‐cation lead bromide perovskite NCs. To study the mixing of the three cations, we first prepared double cation perovskite NCs, after which the third cation was introduced into the crystal lattice, and this process was probed by PL emission. As depicted in Figure 3c, the addition of FAPbBr_3_ NCs into MAPbBr_3_ NCs led to a redshift of the emission peak, suggesting the formation of MA_*x*_FA_1−*x*_PbBr_3_ NCs. Then, upon addition of the CsPbBr_3_ NCs into the double‐cation colloidal solution, the PL emission blue shifted due to the introduction of MA into the crystal lattice, indicating the formation of MA_*x*_FA_*y*_Cs_1−*x*−*y*_PbBr_3_ NCs. The single PL peak of the resulting colloidal solution again corroborates the homogeneous mixing of the three cations in the crystal lattice, otherwise, we would see multiple PL peaks. The cubic shape of the NCs was preserved after the formation of triple‐cation perovskite NCs, and additionally, their size appeared to be rather uniform with an average edge length of ≈9 nm (Figures 3d, e). The HAADF‐STEM image (Figure 3f) shows a uniform contrast of the A‐site columns in the triple‐cation perovskite NCs.

This is the first report demonstrating the preparation of triple A‐cation perovskite NCs by post‐synthetic cation cross‐exchange. Previously, triple‐cation NCs were prepared by a ligand‐assisted reprecipitation approach that produced spherical NCs.^[16]^ In contrast, the post‐synthetic ion‐exchange approach offers shape control based on the pre‐synthesized template NCs. Furthermore, we find that these triple cation NCs exhibit higher long‐term stability compared to organic‐inorganic hybrid LHP NCs that are known to be relatively unstable, as evidenced by UV/Visible absorption measurement at their peak position (see Figure S7). Future studies will investigate the composition‐dependent phase stability of mixed cation LHP NCs. To understand the structural features of the double‐ and triple‐cation lead bromide perovskite NC samples, XRD analysis was performed and the results are depicted in Figure S8 of the Supporting Information. The XRD pattern of CsPbBr_3_ NCs exhibit typical features matching closely with the orthorhombic phase, whereas the XRD patterns of the hybrid perovskite NC samples (MAPbBr_3_and FAPbBr_3_) conform to the cubic phase, and these observation are consistent with previous reports.^[17]^ Interestingly, the XRD pattern of the double‐cation (CsFA) lead bromide NCs (with PL peak cantered at 525 nm) shows the splitting of the peaks in lower 2‐theta range (15 degree) and thus could be better indexed as an orthorhombic phase. Contrary to this, the triple‐cation (MACsFA) lead bromide NCs sample (with emission cantered at 520 nm) evidenced diffraction features matching closely with the cubic phase. Such structural transformation from the orthorhombic to cubic phase could be ascribed to the presence of both MA and FA ions in the NC lattice which heals the lead bromide octahedron distortion of the native lattice (CsPbBr_3_ and mixed cation Cs_*x*_FA_1−*x*_PbBr_3_). The observed structural transformations are indeed interesting, and the detailed investigation will be the subject of future studies.

**Figure 4:**
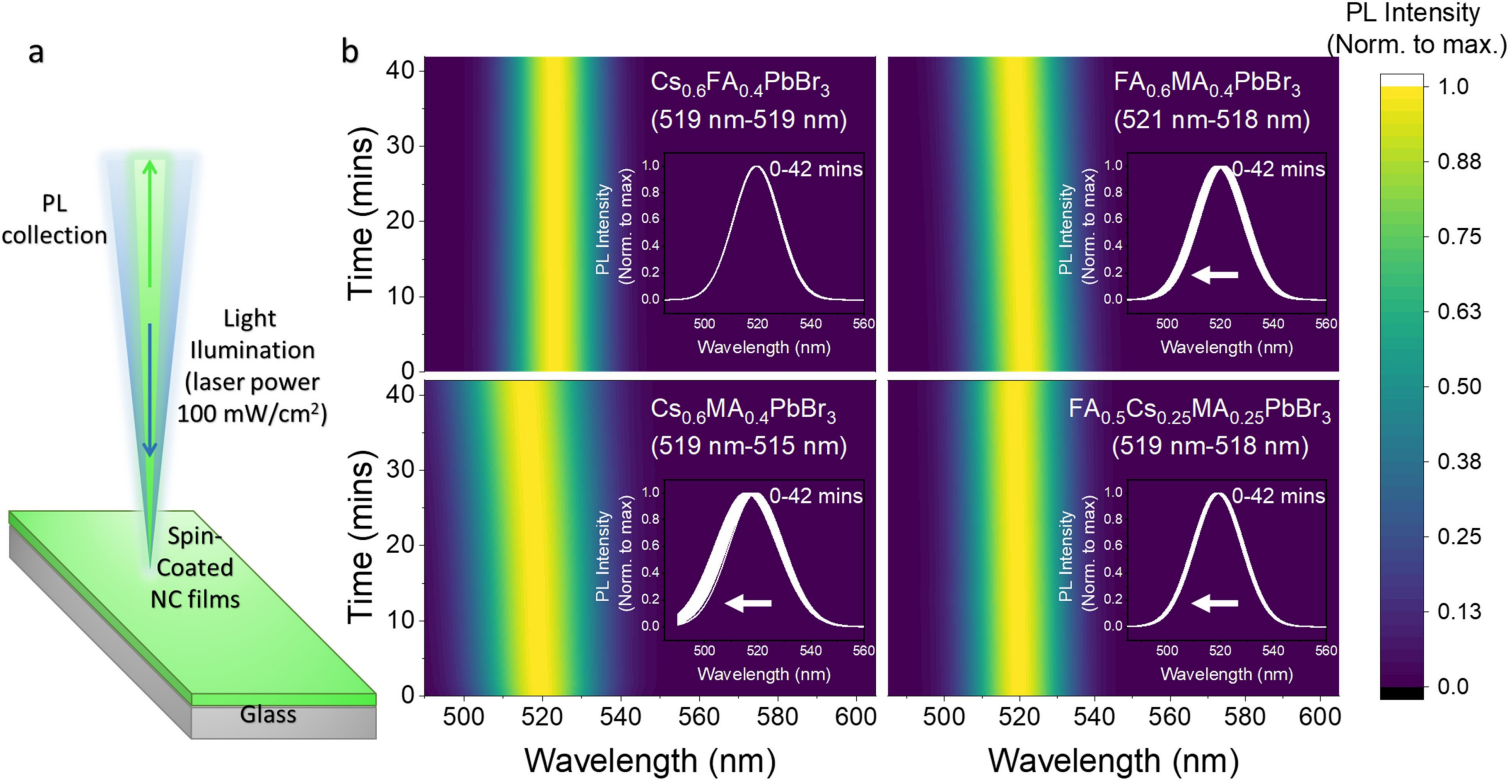
a) Schematic of the hyperspectral microscopy setup used to characterize the PL stability of spin‐coated double and triple perovskite NC films on glass substrates. b) The fitted 2D PL spectral over time (from 0 mins to 42 mins under direct illumination of 400 nm CW laser with a power density of 100 mW cm^−2^) of the double (CsFA, FAMA, and CsMA) and triple (FACsMA) cations perovskite NC films. The inserts show the evolution of the PL spectra over time from 0 to 42 minutes. The compositions are approximated based on the absorption spectra of the pure NCs mixed. Colour scale bar (0–1) is provided on the right side for the intensity of the PL.

It is well‐known that mixed‐halide perovskites exhibit photoinduced halide segregation under light illumination, resulting in the splitting of PL into two distinct peaks corresponding to their individual halides or the PL peak red‐shifting over time, as the photo‐excited carriers relax to the lowest‐band gap phase present.[^15b^, ^18^] To investigate whether or not the A‐cations segregate in the mixed A‐cation NCs, we acquired the PL spectra of the mixed A‐cation NC film under intense light illumination. A simplified schematic of the setup is illustrated in Figure 4a (see Supporting Information for more details). The samples studied here include Br‐based double and triple A‐site cation perovskite NC thin films. The compositions provided in Figure 4 are approximated based on the concentration of the individual NCs mixed to obtain mixed‐cation NCs, assuming that the NCs exhibit the same extinction intensity regardless of their A‐site cation composition. Figure 4b demonstrates the fitted normalized 2D PL intensity map over 42 min with continuous illumination. In all the mixed A‐site cation NC films, no PL splitting was observed. However, the PL peak became broader over time (Figure 4b, see Figure S9 for further discussion on the origin of broadening). On the other hand, the PL spectra of the CsFA perovskite NCs remained unchanged over the course of the measurement (Figure 4b; (data normalized to the maximum PL) and Figure S8 (unnormalized data)), suggesting that no macroscopic phase‐separation occurred. In all the cases, the single PL peaks did not split into multiple peaks, which would have usually been a sign of halide segregation in mixed halide NCs. The unchanged spectral shape clearly indicates that there is no cation phase segregation in these NCs. However, the PL spectra were irreversibly blue‐shifted for samples containing MA in the mixed A‐site, including the CsMA, FAMA and FACsMA samples. This was especially the case for CsMA and FAMA double cation systems, which had a higher fraction of MA present. That is, the PL blue‐shifted by 4 nm for CsMA, and 3 nm for FAMA, whilst the blue‐shift was only 1 nm for the triple cation system. One possible explanation for this irreversible blue shift is related to the removal of the MA cations due to the thermal energy provided by the excitation laser, which would reduce the size of the NCs or contract the lattice since the larger MA cations are removed from the A‐sites. The intensity of the PL also continuously decreased over the 40 min measurement in all cases (Figure S10). This may be due to the removal of A‐site cations lead to the formation of surface traps that reduce the PLQY.

**Figure 5:**
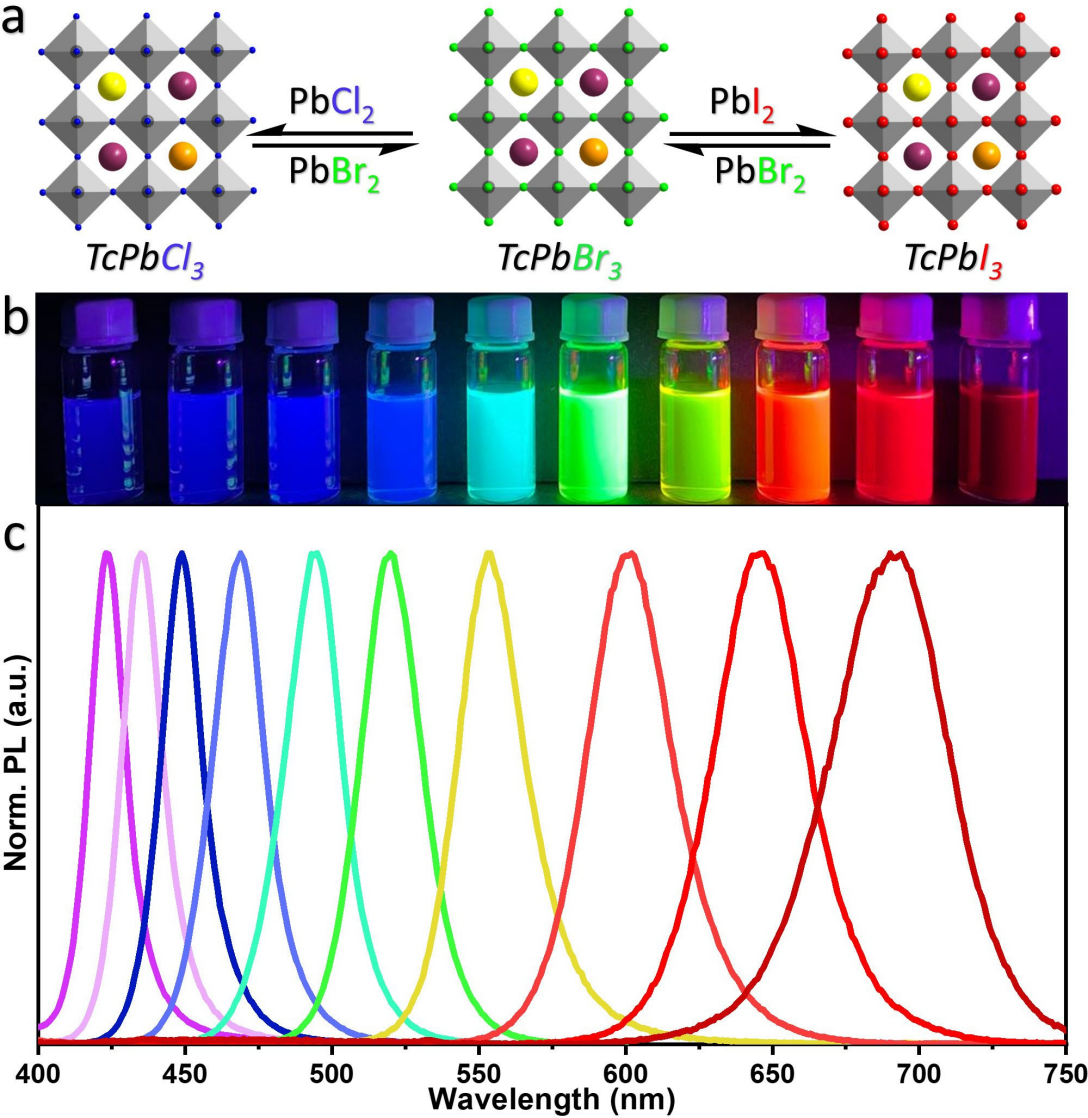
a) Schematic illustration showing the transformation of TcPbBr_3_ into TcPbCl_3_ and TcPbI_3_ NCs through halide ion exchange reaction by the addition of PbCl_2_ and PbI_2_ precursor solutions, respectively. Tc: triple cation (MACsFA) b) Photograph of corresponding colloidal dispersions of colloidal TcPbX_3_ perovskite NCs with different halide (X=Cl, Br, and I) compositions under UV light illumination. c) Normalized PL spectra of the corresponding colloidal NCs.

The optical properties of the triple‐cation perovskite NCs are tunable across the visible spectrum by varying their halide composition, similar to mono‐cation perovskite NCs.^[1a]^ As shown in figure 5, the emission colour of the triple‐cation perovskite NCs is tunable across the visible spectrum of light by applying post‐synthetic halide exchange on TcPbBr_3_ NCs (Tc: triple‐cation). As expected, the PL spectrum emission of TcPbBr_3_ NCs blue‐shifted upon reacting with the PbCl_2_‐ligand solution, while it red‐shifted upon reacting with the PbI_2_‐ligand solution (Figure 5c), and thus the NCs emitting all visible colors are achievable (Figure 5b). However, these triple cation perovskite NCs are difficult to obtain directly from their precursors due to differences in the crystallization conditions of their individual components. The fast A‐site cation cross‐exchange along with fast halide ion exchange enables the preparation of a wide range of new NCs with variable compositions. These results are remarkable because it was previously thought that the activation energy barrier that needs to be overcome for cation exchange is higher than for halide ion exchange.^[7]^ In the case of halide ion cross‐exchange, it was proposed that the shuttling of halide ions between different NCs can be promoted either by the residual halide ions presented in the perovskite NC colloidal dispersions in the form of alkylammonium halides or through desorption of NC surface halides.^[1b]^

**Figure 6:**
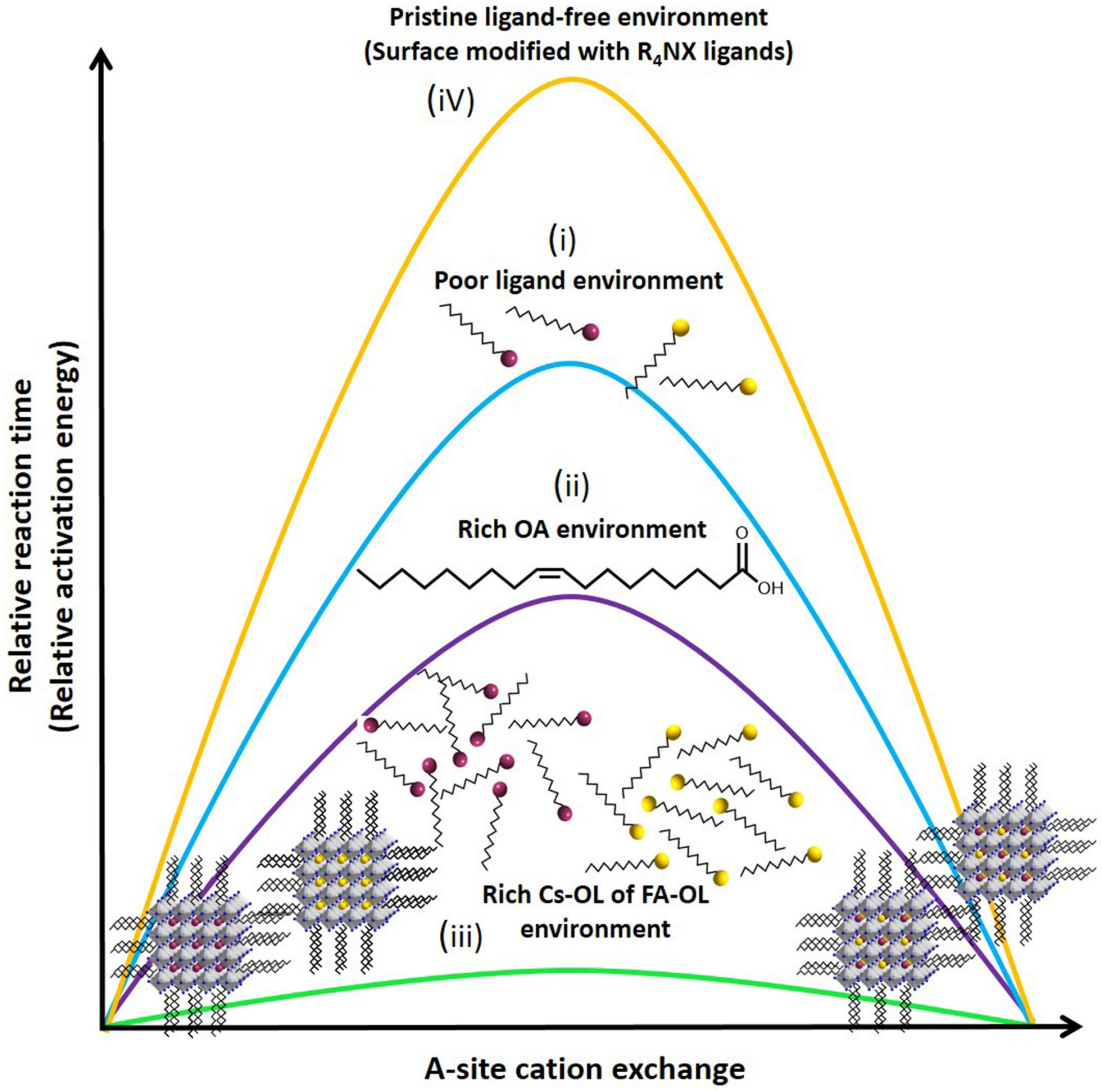
Schematic of relative activation energy barrier for A‐site cation cross‐exchange between purified CsPbBr_3_ and FAPbBr_3_ NCs (washed twice with MeOAc) under different ligand conditions. The schematic is based on the reaction kinetics of the cross‐exchange reaction, which was probed by PL (see Figures S11 and S12). The time required for completion of reaction is directly proportional to activation energy. i) The NCs were purified using antisolvent, ii) oleic acid was added into purified NCs, iii) A‐oleate complexs (Cs‐oleate or FA‐olate) were added into purified NCs, iv) The surface of the NCs were replaced with R_4_NX (DDAB) ligands, meaning that the NCs are completely free of A‐oleate complexes.

To understand the origin of the fast A‐site cation cross‐exchange at room temperature, we ran the reaction on well‐purified CsPbBr_3_ and FAPbBr_3_ NCs (washed twice using methyl acetate (MeOAc) under four different conditions, where we varied the ligands, as illustrated in Figure 5. The four different conditions are i) no further addition of ligands, ii, iii) addition of oleic acid and A‐oleate complex immediately after mixing the NCs, respectively, and iv) the surface of the NCs is modified with didodecyldimethyl ammonium bromide (DDABr)^18]^ (see Supporting Information for experimental details). Although the NCs were well purified, the colloidal suspension still contained at least the minimum amount of ligands to stabilize the NCs and these ligands can assist the cation cross‐exchange. The reaction time can vary based on the amount of ligands left in the solution after washing. So, the amount of MeOAc used for washing the NCs and the number of washing cycles are very important.^[19]^ Therefore, all the control experiments were performed on NCs washed in a similar way. The kinetics of the four reactions were probed by time‐resolved PL measurements (see Figure S11 and S12 for the kinetics of each reaction system). Based on the observed kinetics, the relative activation energy (which is directly proportional to the time required for the reaction to complete) of the cross‐exchange reaction under four reaction conditions is illustrated in Figure 6 for comparison. In the well‐purified NCs, where the residual ligand (A‐oleate, olelylamine (OLA), oleic acid (OA)) concentration is minimal, the cross‐exchange reaction was completed in 1 hr. (Figure S11a), which is much slower compared to the reaction in pristine (i.e. less purified) NCs, where it was completed in 2–3 minutes. This suggests that there is a high kinetic barrier for the cations to shuttle between purified NCs without a significant amount of ligands in them, as illustrated in Figure 6‐i. The complete removal of the parent ligands (OLA, OA, A‐oleate) by ligand exchange with R_4_NX further increased the kinetic barrier and the reaction was not complete even after 24 hrs (Figures 6‐iv and S12). However, the reaction time reduces from 1 hr to 20 min after introducing the OA ligands into the colloidal mixture of purified pristine NCs (Figure 6‐ii and S11b) (note: the addition of OLA has no significant influence). It is most likely that the added OA ligands displace the A‐site cations of the NC surface and form A‐oleate complexes, through which the cation exchange takes place by reducing activation energy (Figure 6‐ii). Yet, the time required for completion of the exchange is higher than that for unpurified pristine NCs (Figure 2a) because of the time needed to solubilize A‐site cations after the addition of OA. These results clarify the role of the residual A‐oleate complexes present in the freshly prepared unpurified colloidal NCs. Further evidence for the critical role of the A‐oleate complex in the cation cross‐exchange came from the dramatic completion of the reaction within a few seconds after the addition of the pre‐synthesized A‐oleate complex (either Cs‐oleate or FA‐oleate) into the mixture of colloidal NCs (Figures S11c, d). This means the A‐oleate complex drastically lowers the activation energy by triggering the shuttling of cations between NCs, as illustrated in Figure 6‐iii. Notably, the addition of an A‐oleate complex (either Cs‐oleate or FA‐oleate) triggers the cross‐exchange. This means that the added Cs‐oleate first replaces FA of the FAPbBr_3_ and then the solvated FA molecules replace the Cs of the CsPbBr_3_, and this process continues until the equilibrium composition is reached.

## Conclusion

In conclusion, we have demonstrated the spontaneous and fast A‐site cation (Cs, MA, and FA) cross‐exchange in halide perovskite NCs at room temperature, and it is as fast as typically found in halide exchange. The controlled experiments revealed that the residual A‐oleate complexes present in the freshly purified pristine NCs facilitate the fast cross‐exchange, in a similar way as the alkylammonium halides do for the halide exchange. We thus prepared double and triple cation perovskite NCs by mixing the respective mono‐cation perovskites. This is the first demonstration of the preparation of triple cation perovskite NCs by cation exchange. The optical properties along with high‐resolution HAADF‐STEM and iDPC‐STEM imaging suggested the homogeneous distribution of the different cations in the mixed perovskite crystal lattice. Unlike halide ions in mixed halide LHP systems, the A‐cations do not segregate under intense light illumination. The band gap of mixed‐cation perovskite NCs is further tunable across the visible range by varying the halide composition. We anticipate that these mixed‐cation perovskite NC systems are expected to be promising for the development of efficient and stable optoelectronic devices, similar to thin film‐based mixed‐cation perovskite systems, which have already shown great promise.

## Conflict of interest

The authors declare no conflict of interest.

1

## Supporting information

As a service to our authors and readers, this journal provides supporting information supplied by the authors. Such materials are peer reviewed and may be re‐organized for online delivery, but are not copy‐edited or typeset. Technical support issues arising from supporting information (other than missing files) should be addressed to the authors.

Supporting Information

## Data Availability

The data that support the findings of this study are available from the corresponding author upon reasonable request.
