# Supplementary material for: Fast A‐Site Cation Cross‐Exchange at Room Temperature: Single‐to Double‐ and Triple‐Cation Halide Perovskite Nanocrystals
Source: Angew Chem Int Ed Engl. 2022 Jul 13;61(34):e202205617. doi: 10.1002/anie.202205617 (PMC9540746; doi:10.1002/anie.202205617)
Supplement: Supplementary file 1 — Supporting Information [file ANIE-61-0-s001.pdf]

Angewandte Chemie
Eine Zeitschrift der Gesellschaft Deutscher Chemiker

Supporting Information

Fast A-Site Cation Cross-Exchange at Room Temperature: Single-to Double- and Triple-Cation Halide Perovskite Nanocrystals

*C. Otero-Martínez, M. Imran, N. J. Schrenker, J. Ye, K. Ji, A. Rao, S. D. Stranks, R. L. Z. Hoye, S. Bals, L. Manna, J. Pérez-Juste, L. Polavarapu**

Experimental Procedures

Materials: Cesium carbonate (Cs_2CO_3, 99,9%), formamidine acetate salt ($CH_4N_2{\cdot}C_2H4O_2$, 99%), methylammonium bromide (CH_3NH_3Br, >99%, anhydrous), lead (II) bromide ($PbBr_2$, >98%), lead (II) iodide (PbI_2, 99%), benzoyl bromide (97%), 1-octadecene (ODE, $C_{18}H_{36}$, 90%) oleic acid (OA, $C_{18}H_{34}O_2$, 90%) and oleylamine (OLA, $C_{18}H_{37}N$, 70%) were purchased from Merck. Methyl acetate (CH_3COOCH_3, 99%) and toluene (C_7H_8, <99.8%) was supplied by Alfa Aesar. All chemicals were used without further purification.

Preparation of a stock solution of cesium-oleate (Cs-OL): In a typical synthesis, 407 mg of Cs_2CO_3 (1.25 mmol) and 1.25 mL of oleic acid (3.5 mmol) were added to 20 mL of 1-octadecene in a 50 mL sample vial. The resulting mixture was heated at 150 ºC under stirring until the salt is completely dissolved. The Cs-oleate complex generally precipitates at room temperature, however, it could be easily dissolved again by continuous stirring at 120 ºC.

Preparation of a stock of formamidinium-oleate (FA-OL): In a typical synthesis, 521 mg of formamidine acetate (5 mmol) was mixed with 20 mL of oleic acid (56 mmol). The resulting mixture was heated at 150 ºC under stirring until the salt was completely dissolved.

Preparation of a stock of different lead halides (PbX_2): In a typical synthesis, the lead halide salt 345 mg of $PbBr_2$ (0.94 mmol) or 433 mg of PbI2 (0.94 mmol)) was added to a mixture of 2.5 mL of OLA (5.3 mmol), 2.5 mL of oleic acid (7.0 mmol) and 25 mL of 1-octadecene in a 50 mL sample vial. The resulting mixture was heated at 150 ºC under continuous stirring until the salt was completely dissolved.

Preparation of OA-Toluene, CsOL-Toluene and FAOL-Toluene solutions for kinetic studies. All the ligand solutions in toluene have same ligand concentration. For OA-Toluene solution, 50 µL of OA were diluted into 950 µL of toluene. For the oleate-toluene solutions first, CsOL and FAOL solutions 0.25 M in OA were prepared by dissolving the corresponding mass of formamidinium acetate and cesium acetate in OA at 125 ºC under stirring. The CsOL-Toluene and FAOL-Toluene solutions employed for the kinetic studies were obtained by dissolving 50 µL of the corresponding oleate solution previously prepared in 950 µL of toluene.

Preparation of PbI_2 precursor solution for halide exchange. 0.1844 g of PbI_2 (0.4 mmol), hexane (10 mL), oleylamine (0.4 mL) and oleic acid (0.4 mL) were combined in a 20 mL vial. The resulting mixture was heated at 50 ºC under continuous stirring overnight to completely dissolve the salt.

Preparation of $PbCl_2$ precursor solution for halide exchange. 0.1112 g of $PbCl_2$ (0.4 mmol), hexane (10 mL), oleylamine (0.8 mL) and oleic acid (0.8 mL) were combined in a 20 mL vial. The resulting mixture was heated at 50 ºC under continuous stirring overnight to completely dissolve the salt.

Synthesis of $CsPbBr_3$ and $CsPbI_3$ perovskite NCs: The synthesis is carried out using the previously reported hotplate approach.[1] In a typical synthesis, 6 mL of the corresponding PbX_2 precursor solution in a 20 mL glass vial was heated on a hot-plate until the temperature of the precursor solution reaches 175 ºC in the case of

$CsPbBr_3$ and 150 ºC for $CsPbI_3$, and then, 400 µL of the pre-heated Cs-OL stock solution was swiftly injected into it under vigorous stirring (1,000 rpm). After 5 s, the vial cooled in an ice-water bath to quench the reaction. Subsequently, the thus obtained colloidal dispersion was purified by centrifugation at 8000 rpm for 10 min. Then, the supernatant was discarded to remove the unreacted precursors and ligands and the pellet was redispersed in 5 mL of toluene. The colloidal dispersion was centrifuged again at 5,000 rpm for 8 min to remove largest particles in the sediment.

Synthesis of $FAPbBr_3$ and $FAPbI_3$: In a typical synthesis, 6 mL of the corresponding PbX_2 precursor solution in a 20 mL glass vial was heated on a hot-plate until the temperature of the precursor solution reaches 175 ºC in the case of $FAPbBr_3$ and 150 ºC for $FAPbI_3$, then, 2.5 mL of the pre-heated FA-OL stock solution was swiftly injected into it under vigorous stirring (1,000 rpm). After 5 s, the vial was removed from the hot-plate and placed it in an ice-water bath to quench the reaction. $FAPbBr_3$The crude solution was purified following the same procedure as described for $CsPbBr_3$ and $FAPbBr_3$ NCs. FAPbI3 crude solution was purified also by centrifugation (8000 rpm, 10 min). Then, the pellet was redispersed in toluene and the colloidal solution was centrifuged again (3500 rpm, 5 min) to remove the largest particles in the sediment.

Synthesis of $MAPbBr_3$ NCs: $MAPbBr_3$ NCs were synthesized by ligand assisted reprecipitation method (LARP). In a typical synthesis, $PbBr_2$ (0.4 mmol), CH_3NH_3Br (0.32 mmol), oleylamine (0.1 mL), and oleic acid (1 mL) were co-dissolved in DMF (10 mL) in a 20 mL glass vial. The salts dissolve immediately without heating or sonication. On the other hand, 10 mL of toluene in a 20 mL vial was heated on a hot-plate until rise 40 ºC under stirring (1,000 rpm), then, 1 mL of the DMF mixture was swiftly injected into it. The solution color turned yellow immediately and it was removed from the hot-plate and placed it in an ice-water bath to quench the reaction. Before purification, it was necessary to induce aggregation by mixing equal volumes of the $MAPbBr_3$ crude solution and methyl acetate. After that, the $MAPbBr_3$ NCs were purified following the same procedure as described for $CsPbX_3$ NCs.

Synthesis of $FA_xCs_{1-x}PbI_3$, $FA_xCs_{1-x}PbBr_3$, $MA_xFA_yCs_{1-x-y}PbBr_3$ NCs by cation exchange: In a typical synthesis, colloidal solutions of $CsPbX_3$, $FAPbX_3$ and $MAPbBr_3$ NCs dispersed in toluene were mixed in different ratios to produce the desired Cs/FA/MA stoichiometry. Before mixing, the absorption spectra of the individual samples were measured, and the concentration was adjusted so that each solution had a similar optical density near the band edge. The mixture was kept stirring (500 rpm) for 5 min at room temperature and the reaction was monitored by photoluminescence spectroscopy.

Kinetics studies: Kinetic studies of different parameters and exchange reactions were carried out in the same way. In a cation or halide exchange reaction, different volumes of colloidal dispersions of the corresponding NCs synthesized previously were mixed under stirring (500 rpm). Before starting the reaction, the absorption spectra of the mono cation and mono halide samples were measured, and the concentration was adjusted so that each solution had a similar optical density neat the band edge. The reaction was monitored by photoluminescence spectroscopy along time. The NCs and measurement parameters employed for each study are described in table 1. For ligands effect study in cation exchange reaction, the colloidal solution in toluene was washed with MeOAc employing proportions 1:1. Then, the mixture was centrifuged (11000 rpm, 15 min) and the sediment was

redispersed in toluene. For rich ligand environment conditions, 100 µL of the corresponding ligand-toluene solutions were added after mixing the NCs.

Table 1. Description of different precursors, solvent, volumes of precursor, reaction temperature, excitation wavelength employed, and final composition obtained for different kinetic studies carried out.

Reaction studied	Precursor 1	Precursor 2	Volume of precursor 1	Volume of precursor 2	Reaction temperature	Excitation wavelength	Final composition
Normally A-cation exchange conditions (Figure 2a)	$CsPbI_3$	$FAPbI_3$	1 mL	1 mL	RT	550 nm	$Cs_xFA_{1-x}PbI_3$
Halide exchange (Figure 2b)	$CsPbI_3$	$CsPbBr_3$	3 mL	1 mL	RT	400 nm	$CsPbBr_xI_{3-x}$
A-cation + Halide exchange (Figure 2c)	$CsPbI_3$	$FAPbI_3$	3 mL	1 mL	RT	400 nm	Cs_xFA_{1-x} $PbBr_yI_{3-y}$
A-cation exchange in surface modified conditions (Figure6)	DDABr capped $CsPbBr_3$	Cs_xFA_{1-x} $PbBr_3$			RT		Cs_xFA_{1-x} $PbBr_3$ and $CsPbBr_3$
A-cation exchange in purified NCs (Figure 6)	$CsPbBr_3$ washed twice with MeOAc	$CsPbBr_3$ washed twice with MeOAc	1 mL	1 mL	RT	400 nm	Cs_xFA_{1-x} $PbBr_3$
A-cation exchange in rich OA environment (Figure 6)	$CsPbBr_3$ washed twice with MeOAc	$FAPbBr_3$ washed twice with MeOAc	1 mL	1 mL	RT	400 nm	Cs_xFA_{1-x} $PbBr_3$
	+ OA-toluene addition		+ 100 µL				
A-cation exchange in rich A-OL environment (Figure 6)	$CsPbBr_3$ washed twice with MeOAc	$FAPbBr_3$ washed twice with MeOAc	1 mL	1 mL	RT	400 nm	Cs_xFA_{1-x} $PbBr_3$
	+ CsOL-toluene or FAOL-toluene addition		+ 100 µL CsOL-toluene / 100 µL FAOL-toluene				

Synthesis of DDABr capped $CsPbBr_3$ NCs: The synthesis of $CsPbBr_3$ NCs and subsequent ligands exchange reactions were performed following previously reported methods with some modification.[2] Briefly, the synthesis of starting NCs was performed in air, in a vial (20mL) on a hot plate equipped with a thermocouple and a magnetic stirrer (600rpm). The Cs-Pb-Oleate stock solution (1.50 mL, separately prepared by combining $Pb(CH_3COO)_2$ .3H$_2$0 (760 mg), Cs_2CO_3 (160 mg) and oleic acid (15.00 mL) in a 25 mL three-neck flask and degassed on a Schlenk line at 90 °C for 3h) was loaded into a vial along with the DDA stock solution (1.50mL) and 1-octadecene (9 mL). The mixture was heated to 70 °C at which point the benzoyl bromide stock solution (0.55mL) was rapidly injected. After 60 seconds, the reaction vial was cooled by plunging it into a water bath. The crude NC solution (3.00 mL) was then mixed with a toluene solution of DDABr (2 mL, 25 mM) and washed with ethyl acetate (15 mL). The NCs were separated by centrifugation at 6000 rpm, re-dispersed in a toluene solution of DDAB and PhEABr (1 mL, 4 mM), vortexed the mixture for 5 min and thereafter washed a second time with ethyl acetate (6 mL). The NCs were again separated by centrifugation at 6000 rpm, re-dispersed in a toluene solution of DDAB (1 mL, 2 mM), and washed a third time with ethyl acetate (6 mL). The NCs were once more separated by centrifugation and re-dispersed in toluene. Finally, the NCs dispersion was centrifuged at 10000 rpm for 5 minutes and the colloidally unstable fraction of the sample was discarded.

The $FAPbBr_3$ NCs used for the control experiment reported in Figure S9 were prepared by using the above-mentioned method. The crude solution of NCs was precipitated with ethyl acetate with a ratio of 1 to 3 (crude solution to ethyl acetate). The NCs dispersion was centrifuged at 6000 rpm for 5 minutes and the colloidally unstable fraction of the sample was discarded. Colloidal dispersions of both samples (DDABr capped $CsPbBr_3$

and purified FA_xPbBr_3) were mixed together under ambient conditions. Optical absorption and PL spectra of the mixture recorded over the period of time are reported in Figure S9.

Synthesis of $MA_xFA_yCs_{1-x-y}Pb(Cl/Br)_3$ and $MA_xFA_yCs_{1-x-y}Pb(Br/I)_3$ by halide exchange. In a typical synthesis, the 5 mL of $MA_xFA_yCs_{1-x-y}PbBr_3$ colloidal solution synthesized previously was gently heated to 40 ºC. Then, different volumes of the corresponding oleylammonium halide solution were injected based on the desired halide composition. The solution was kept under stirring and heating for 10 min.

Characterization: UV-Vis extinction spectra were carried out using a Cary-60 UV-Vis spectrophotometer (Agilent). Photoluminescence spectra were obtained with a Cary Eclipse Fluorescence Spectrophotometer (Agilent). Quartz cuvettes with an optical path length of 1 cm were used for both optical analyses. Transmission electron microscopy (TEM) images were obtained with a JEOL JEM 1010 transmission electron microscope operating at an acceleration voltage of 100 kV. High resolution HAADF-STEM and iDPC-STEM images were acquired with a probe-corrected cubed Thermo Fisher Scientific Themis Z Microscopeoperating at 300 kV and an electron beam current of 2 pA (electron dose < 400 $e^-/\AA^2$) with a probe semi-convergence angle of 21 mrad. The lattice parameter analysis was performed using StatSTEM.[3] The location of all atomic columns was determined by fitting Gaussian functions to these columns. Time-resolved photoluminescence spectra were obtained using a FluoroMax-3 (Horiba Jobin Yvon) spectrophotometer. The PL decay traces were measured by exciting the samples at 287 nm using <1.2 ns laser diode.

Hyperspectral microscope characterization: Wide-field, hyperspectral microscopy measurements were carried out using a Photon etc. IMA system. For all measurements, ×100 air, chromatic aberration corrected objective lenses from Olympus (MPLFN and MPLAPON) were used. All samples were prepared by spin-coating NCs solution (1500 rpm for 30 seconds) on pre-cleaned glass substrates (glass substrates are cleaned by ultrasonicating in acetone and iso-propanol for 15 mins and plasma treated in oxygen for 10 mins.) A 405-nm continuous wave laser was used for luminescence excitation. Then 50-W halogen lamps were used for transmission and reflection measurements. The excitation laser was filtered by a dichroic mirror. The lamp light used for reflection measurements travels through the objective to the sample. The lamp used for transmission measurements was focused on the sample by a condenser lens from below the sample and is collected by the objective lens. The emitted/transmitted/reflected light from the sample was incident on a volume Bragg grating, which splits the light spectrally onto a CCD camera. The detector was a 1,040 × 1,392 resolution silicon CCD camera kept at 0 °C with a thermoelectric cooler and has an operational wavelength range of 400–1,000 nm. By scanning the angle of the grating relative to the incident light, the spectrum of light coming from each point on the sample could be obtained. For Br-based NCs, we collected the signal from 485 nm to 600 nm.

For calibration of the system to extract the absolute number of photons at each point, a two-step process was used for each objective lens used. First, a calibrated white light lamp from Ocean Optics was coupled into an integrating sphere. The objective lens was also coupled into the integrating sphere. Comparing the measured spectrum of the lamp at each point to the calibrated spectrum gives the relative sensitivity of the system both spectrally and spatially. Second, a 657-nm laser was coupled directly to the microscope by an optical fibre. The power of the laser was measured precisely at the output of the fibre using a power meter before coupling to the

objective lens. Measuring the laser on the system allows direct conversion between number of counts and photons at this wavelength. Combining this absolute calibration with the relative calibration from the calibrated white light lamp and integrating sphere allows absolute calibration across the spectrum at each point of the sample. For all measurements in this experiment, 100 mW/cm2 power density is used to mimic the power of one sun to investigate the PL stability of the NC films.

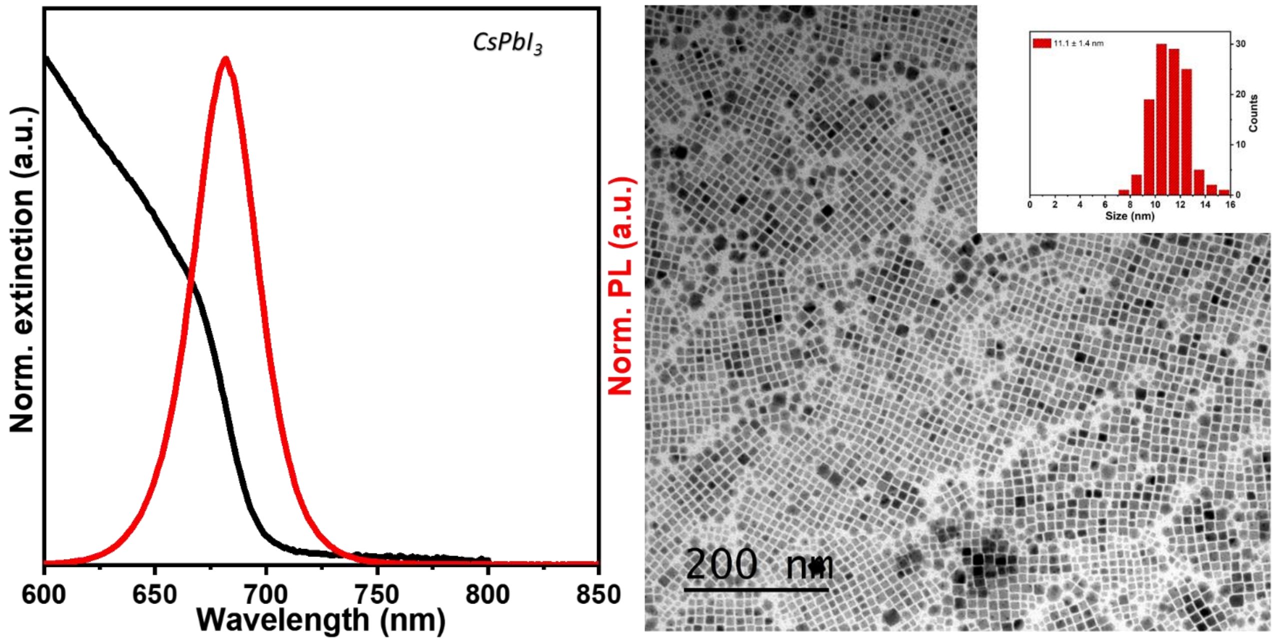

Figure S1. (a) Normalized extinction (black) and photoluminescence (red) spectra of a colloidal dispersion of $CsPbI_3$ NCs. (b) Representative TEM image of $CsPbI_3$ cubic nanocrystals and size distribution histogram in the inset.

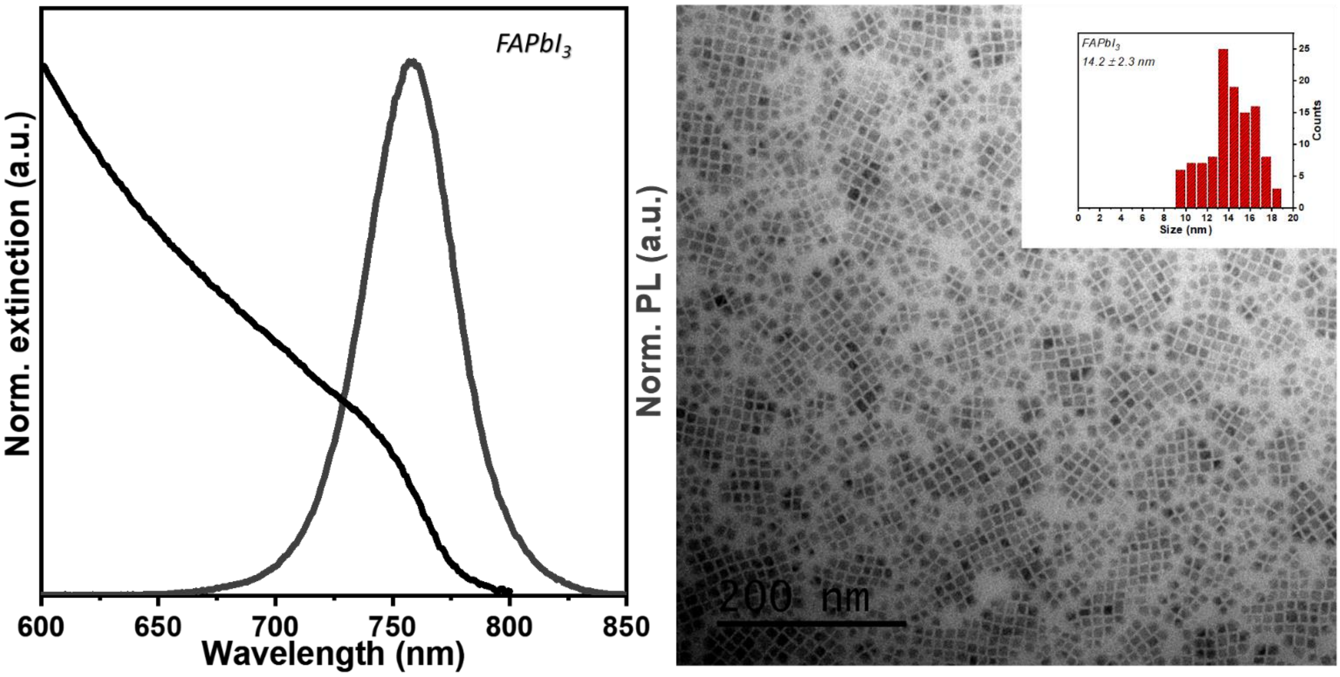

Figure S2. (a) Normalized extinction (black) and photoluminescence spectra (grey) of a colloidal dispersion of $FAPbI_3$ NCs. (b) Representative TEM image of $FAPbI_3$ cubic NCs and size distribution histogram in the inset.

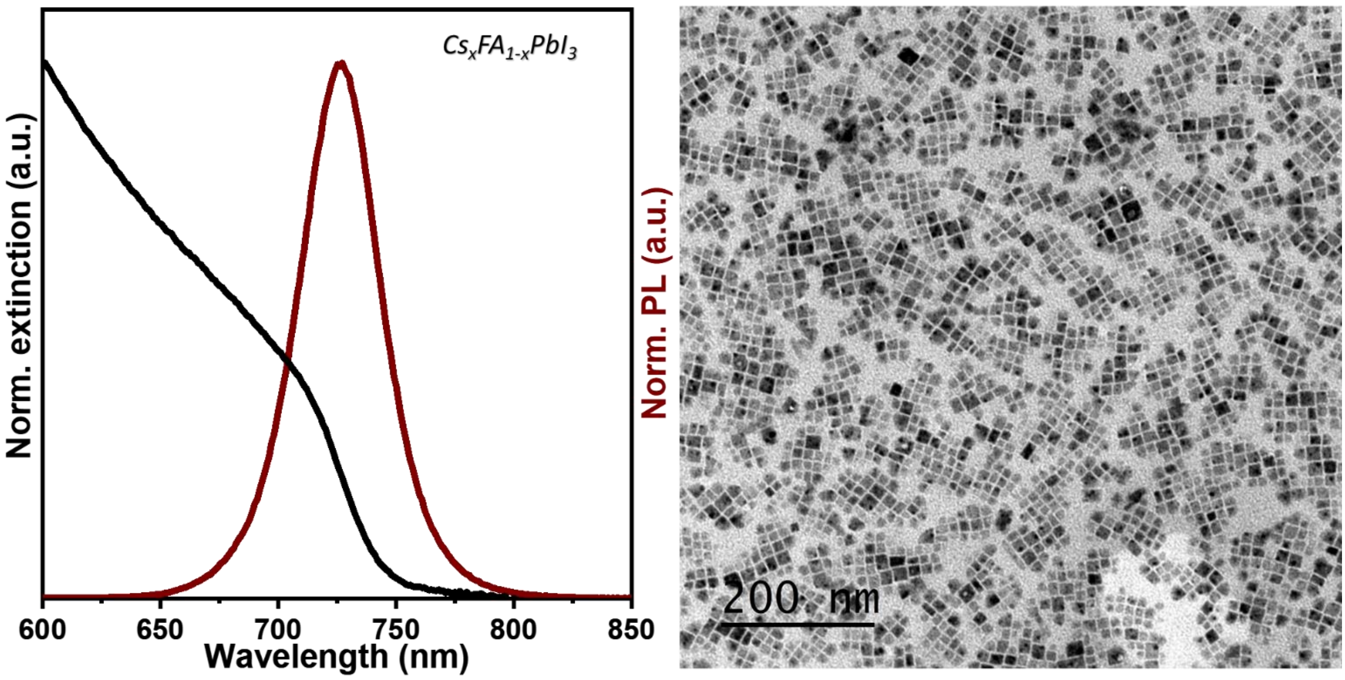

Figure S3. (a) Normalized extinction (black) and photoluminescence spectra (brown) of a colloidal dispersion of $Cs_xFA_{1-x}PbI_3$ NCs. (b) Representative TEM image of $Cs_xFA_{1-x}PbI_3$ cubic NCs.

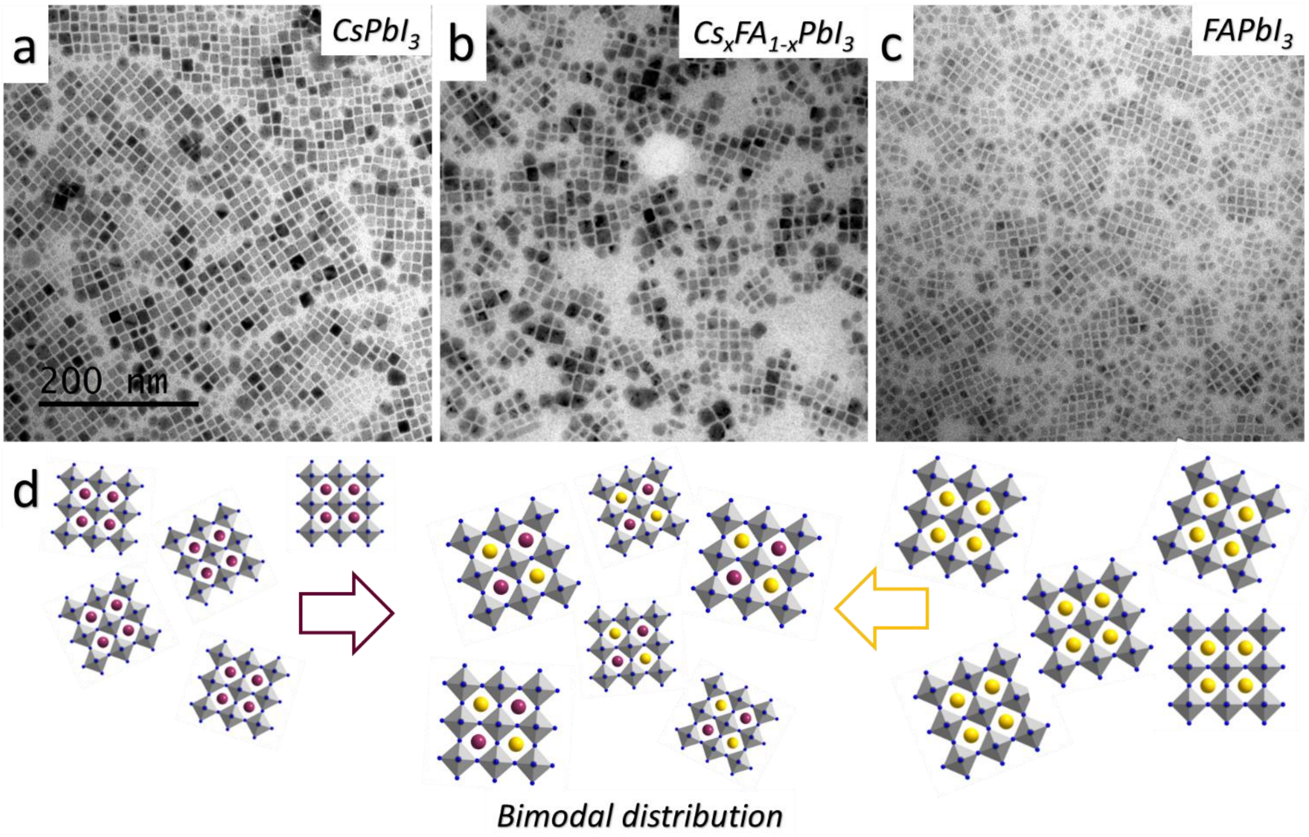

Figure S4. (a-c) Representative TEM image of $CsPbI_3$ (a), $Cs_xFA_{1-x}PbI_3$ (b) and $FAPbI_3$ cubic NCs. (d) Scheme of bimodal size distribution obtained after cation exchange reaction between $CsPbI_3$ and $FAPbI_3$ NCs of different size.

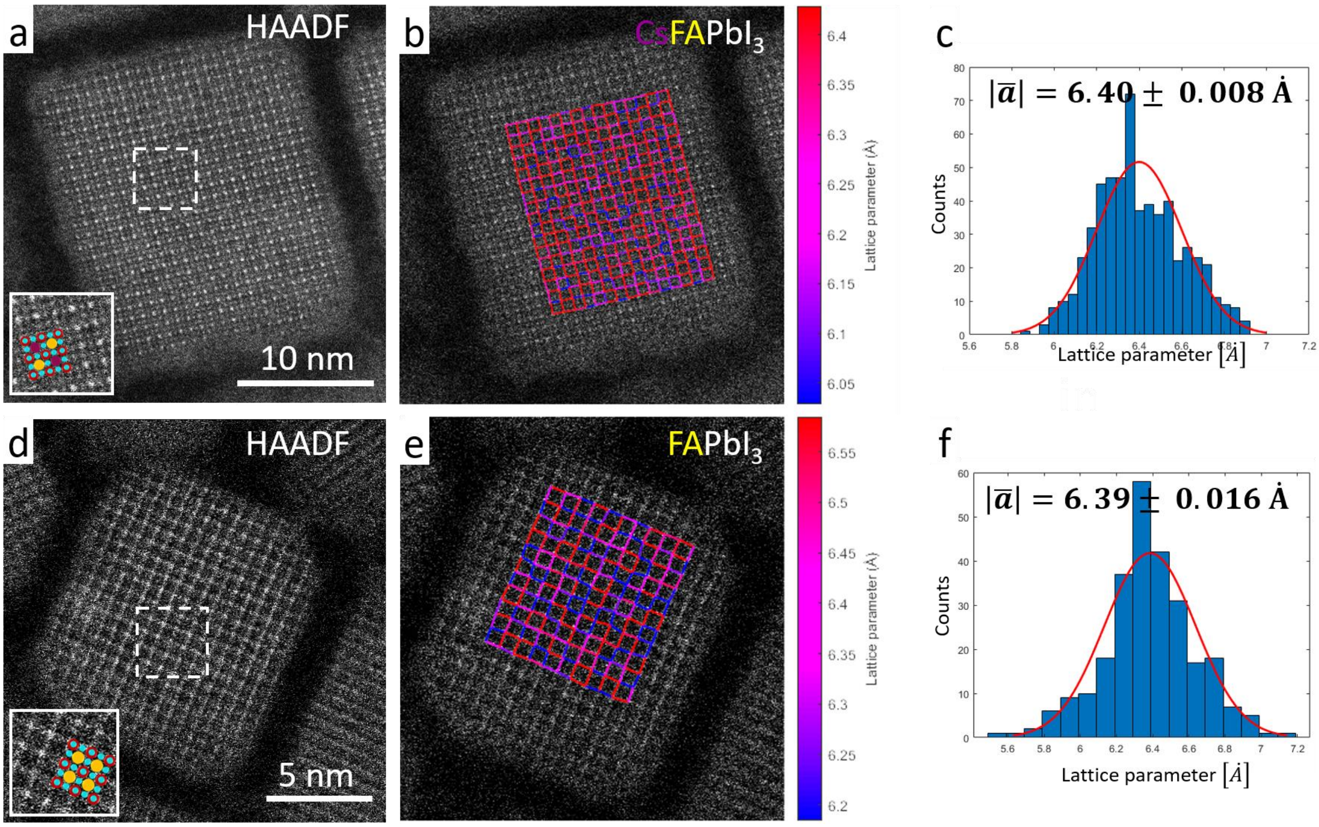

Figure S5. HAADF STEM images of $CsFAPbI_3$ (a) and $FAPbI_3$ (d) NCs. Neighbour column distance mapping of the corresponding NCs using StatSTEM is shown in panel (b) and (e), respectively. The lattice parameter distribution of the maps in panels (b,e) is depicted in panel (c,f). The error corresponds to the standard error on the mean value. The overlay in the insets in panels (a,d) indicates the atom column position of Cs (purple), FA (yellow), Pb (red) and the halide (blue) in the perovskite structure. The Cs and FA cations are randomly mixed.

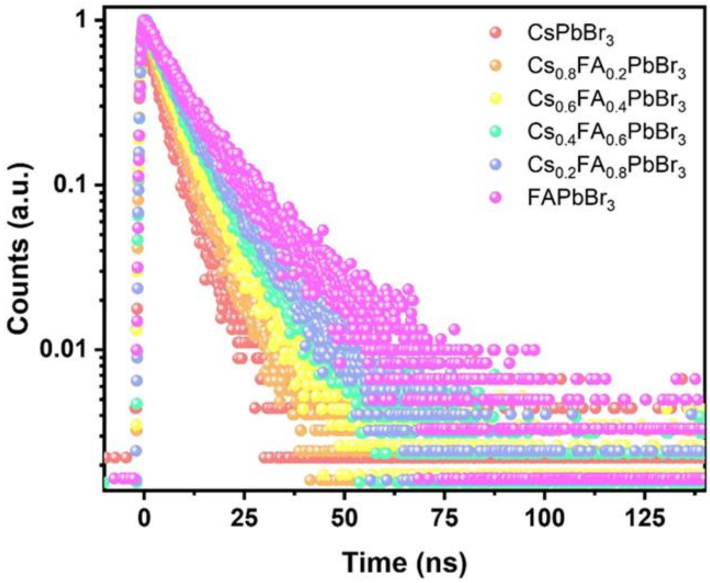

Figure S6. Time-resolved PL decays of $CsPbBr_3$, $FAPbBr_3$ and double A-cation $Cs_xFA_{1-x}PbBr_3$ NCs of different FA/Cs ratios. The compositions is approximated based on the concentration of the individual NCs mixed to obtain mixed-cation NCs, assuming the NCs to exhibit the same extinction intensity regardless of their A-site cation composition.

Table S1. PLQY and time resolved PL decay times (biexponential fitted) for $CsPbBr_3$, $FAPbBr_3$ and mixed A-cation $Cs_xFA_{1-x}PbBr_3$ NCs.

	A_1	τ_1 (ns)	A_2	τ_2 (ns)	$\tau_{average}$ (ns)	PLQY (%)
**$CsPbBr_3$**	0.46	2.39	0.52	6.54	5.52	52.26
**$FA_{0.2}Cs_{0.8}PbBr_3$**	0.83	5.11	0.09	14.11	7.15	77.09
**$FA_{0.4}Cs_{0.6}PbBr_3$**	0.80	5.60	0.16	13.86	8.39	73.60
**$FA_{0.6}Cs_{0.4}PbBr_3$**	0.77	6.53	0.19	15.21	9.71	74.58
**$FA_{0.8}Cs_{0.2}PbBr_3$**	0.80	7.16	0.16	17.82	10.67	73.67
**$FAPbBr_3$**	0.75	8.65	0.20	21.27	13.70	70.82

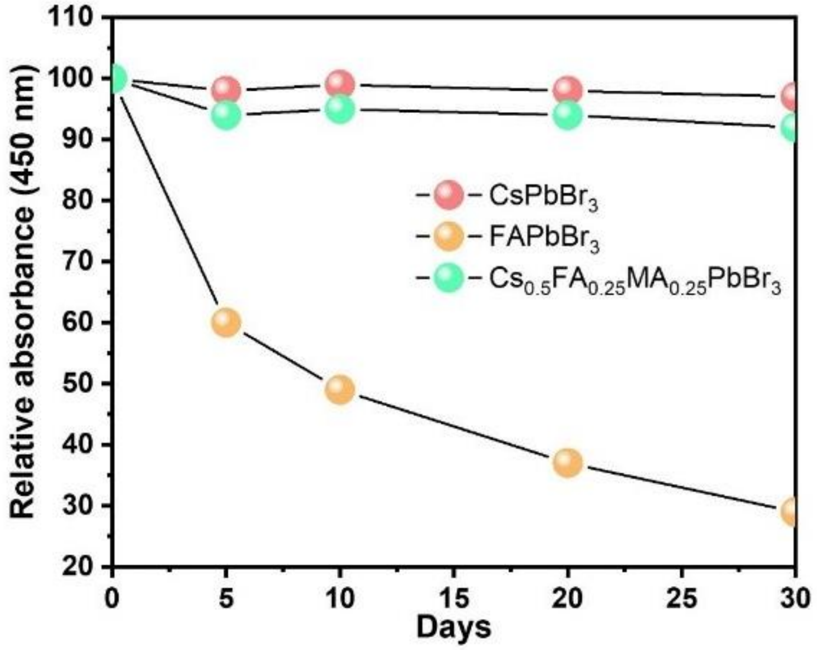

Figure S7: Relative absorbance of $CsPbBr_3$, $FAPbBr_3$, and triple cation ($Cs_{0.5}FA_{0.25}MA_{0.25}PbBr_3$) perovskite NCs over a time of 30 days.

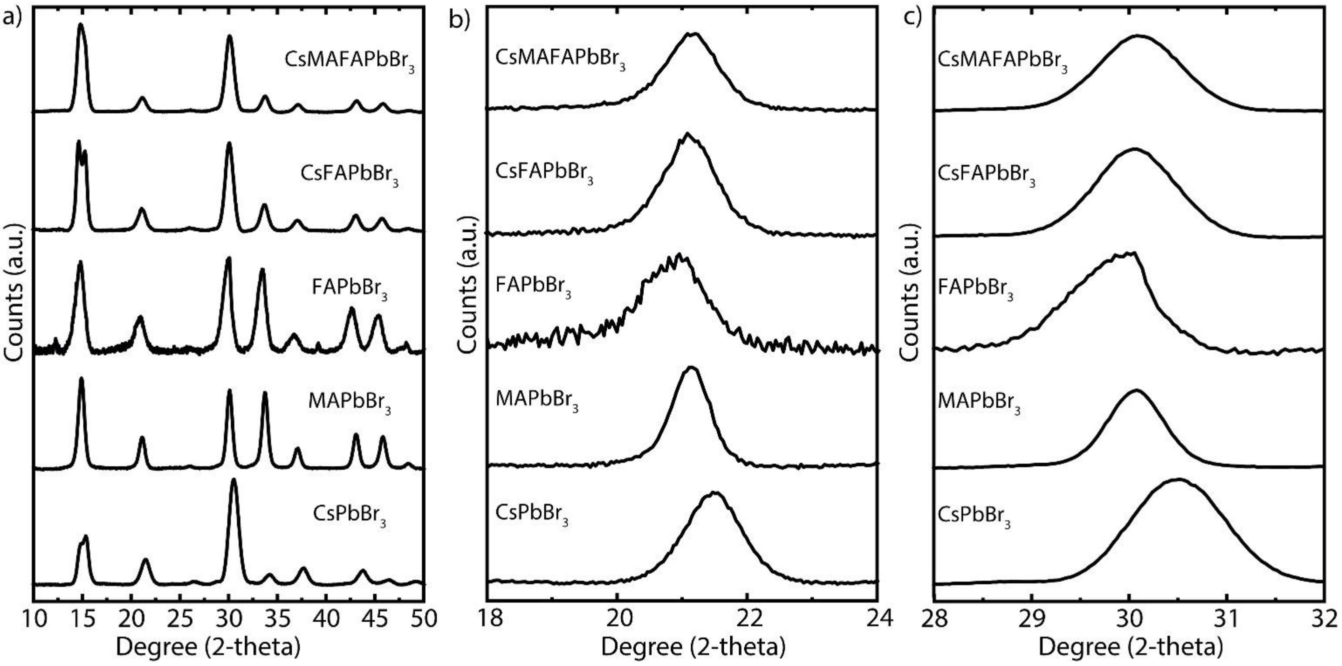

Figure S8. Comparison of XRD patterns of $CsPbBr_3$, $MAPbBr_3$, $FAPbBr_3$, $Cs_xFA_{1-x}PbBr_3$ and $Cs_xMA_yFA_{1-x-y}PbBr_3$ NCs.

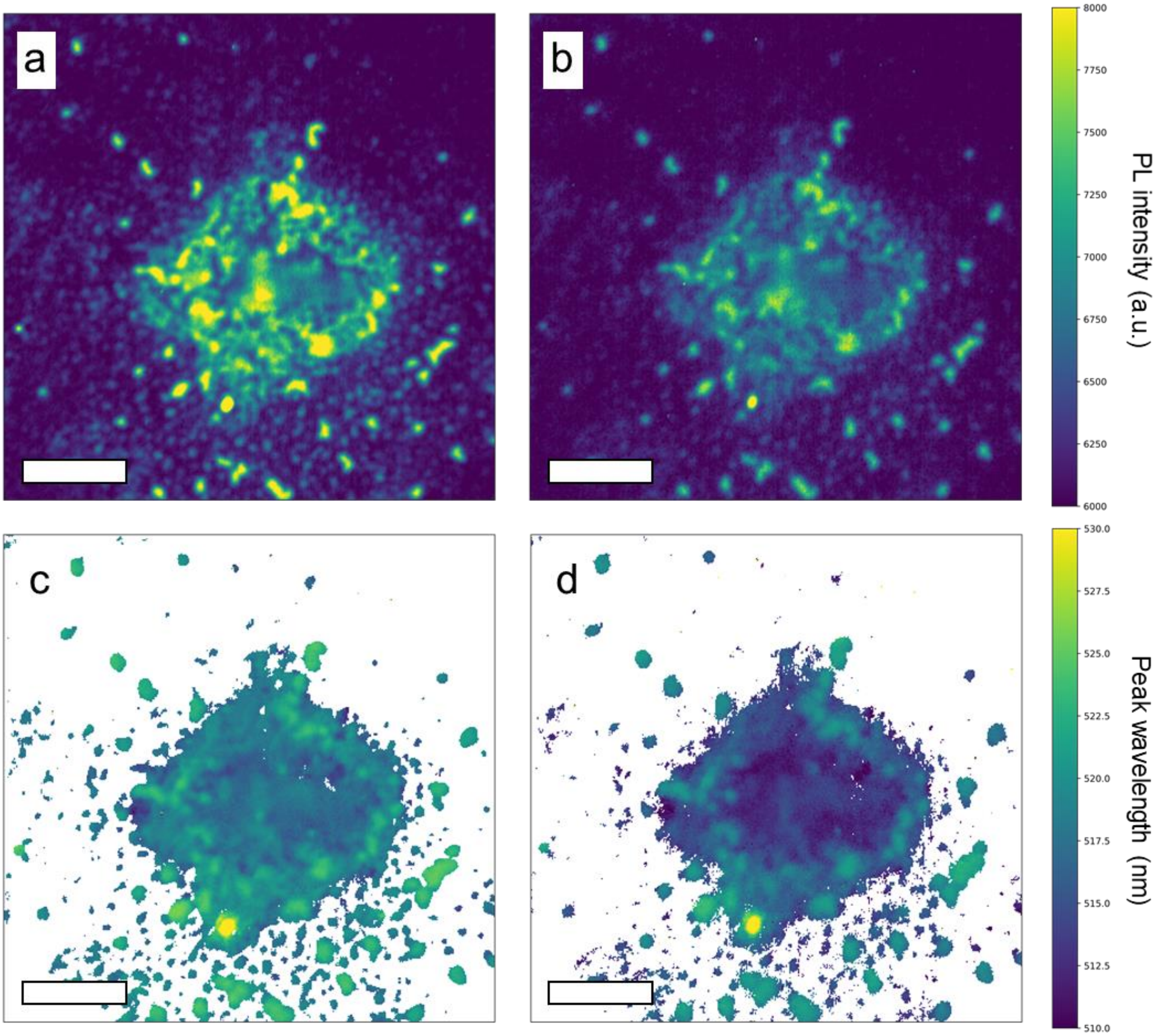

Figure S9. PL intensity maps of CsMA perovskite NC films at 0 min (a) and 40 min (b) of continuous illumination and their corresponding fitted peak wavelength maps (c,d). Scale bar is 5 μm. The broadening of PL peak is likely caused by the differences in blue-shifting speeds of big or small clusters of NCs on the film. Small clusters of NCs are shifting faster than bigger clusters because each NC received higher photon injections (so cation evaporates faster). With a smoother film, it is highly likely that the overall PL spectrum will be blue shifting uniformly without being broadened.

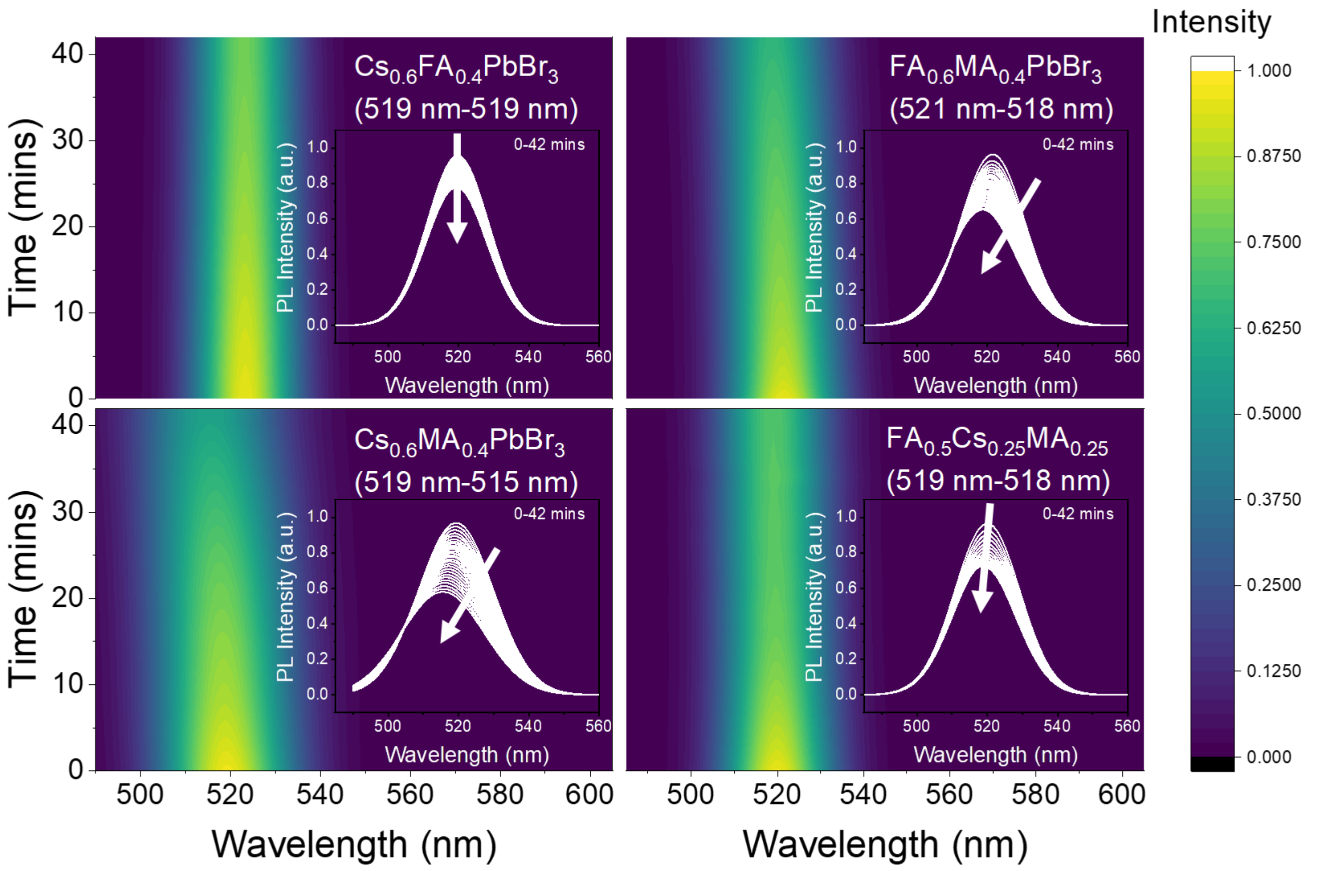

Figure S10. The unnormalized PL spectra. The fitted unnormalized 2D PL spectral over time (from 0 mins to 42 mins under direct illumination of 400 nm CW laser with a power density of 100 mW/cm^2) of the double (CsFA, FAMA, and CsMA) and triple (FACsMA) cations perovskite NC films. The inserts show the evolution of the PL spectra over time from 0 to 42 mins.

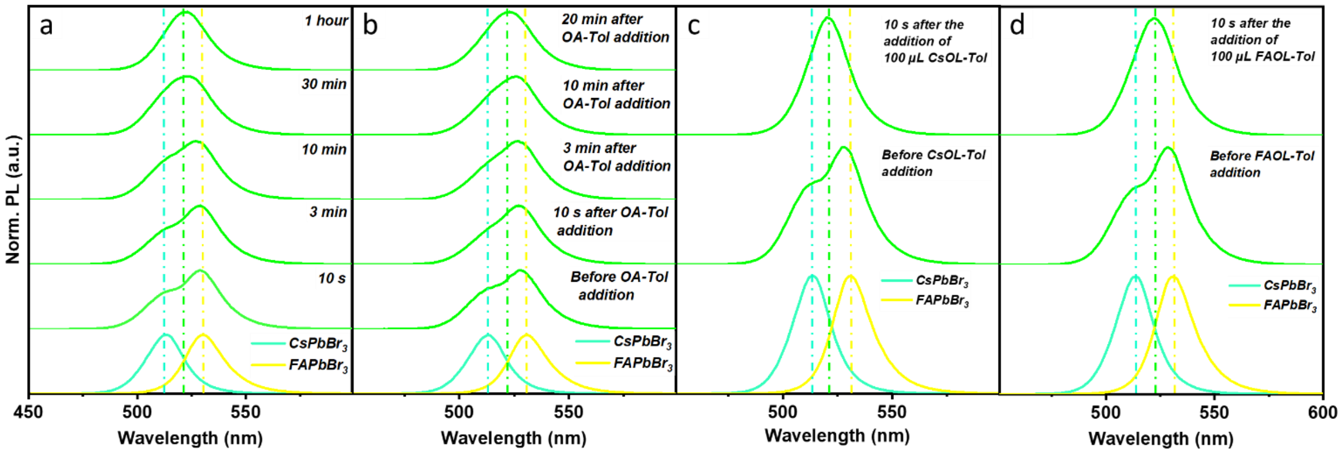

Figure S11. Time-dependent PL measurements of $CsPbBr_3$ and $FAPbBr_3$ A-cation exchange reaction under different environment conditions: poor ligand conditions (a), OA rich conditions (b), rich Cs-OL conditions (c) and rich FA-OL conditions (d).

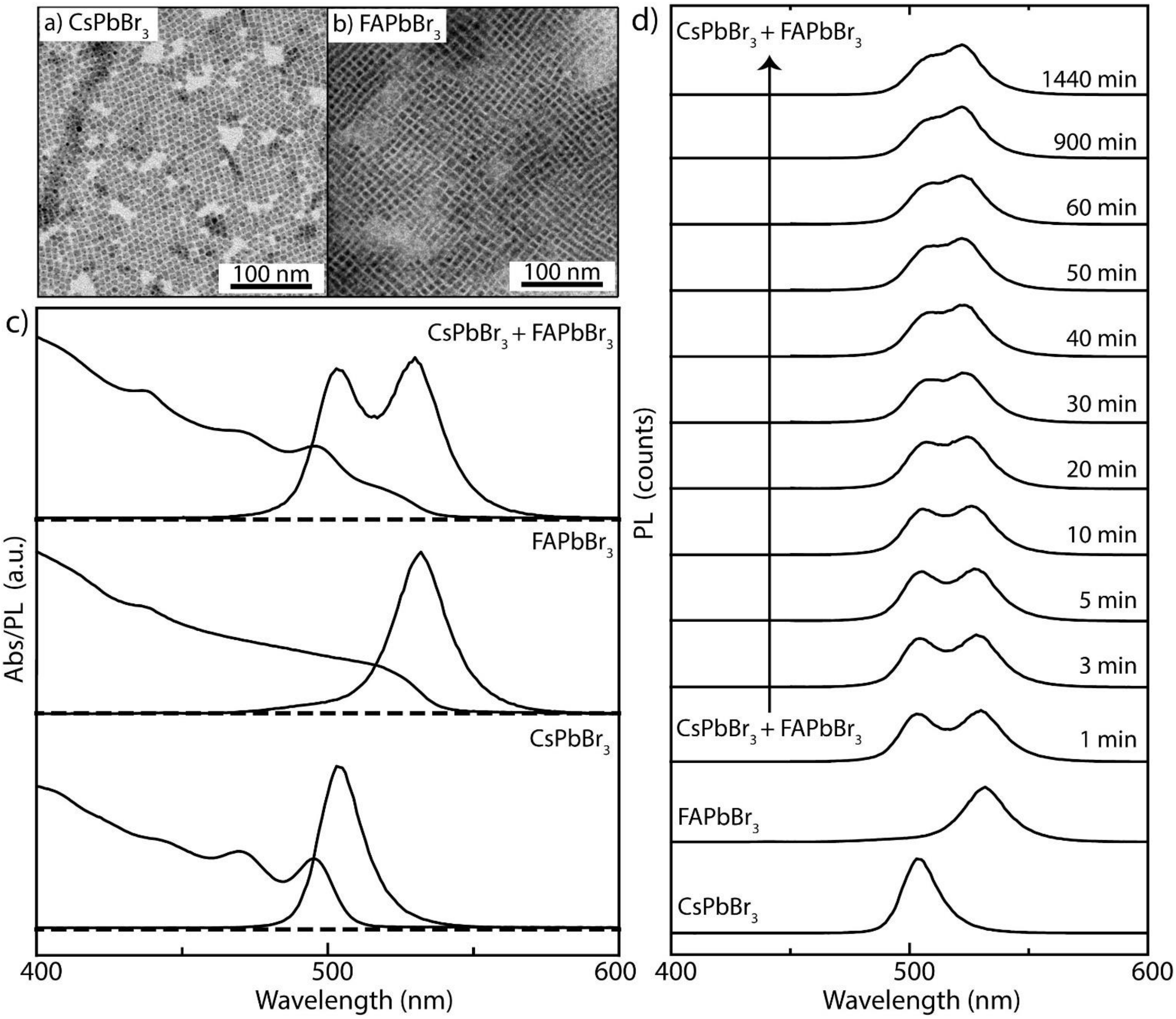

Figure S12. (a) TEM images of DDABr capped $CsPbBr_3$ (a) and $FAPbBr_3$ NCs (b) employed for the kinetic study. (c) Extinction and PL spectra of colloidal dispersions of capped DDABr capped $CsPbBr_3$ NCs, $FAPbBr_3$ NCs, and their mixture immediately after mixing (see experimental section). The two PL peaks in the mixture are correspond to two different species in the reaction mixture. (d) The evolution of the PL spectra after mixing the colloidal dispersions of DDABr capped $CsPbBr_3$ and $FAPbBr_3$ NCs with respect to their individual PL spectra. The multiple peaks in the PL spectra indicate that the cation cross-exchange didn't complete even after 1440 min, meaning that the DDABr protection on the $CsPbBr_3$ NC surface prevents the cation cross-exchange.

References

[1] C. Otero-Martínez, D. García-Lojo, I. Pastoriza-Santos, J. Pérez-Juste, L. Polavarapu, *Angew. Chem. Int. Ed.* **2021**, *60*, 26677-26684.

[2] aM. Imran, P. Ijaz, D. Baranov, L. Goldoni, U. Petralanda, Q. Akkerman, A. L. Abdelhady, M. Prato, P. Bianchini, I. Infante, L. Manna, *Nano Letters* **2018**, *18*, 7822-7831; bM. Imran, P. Ijaz, L. Goldoni, D. Maggioni, U. Petralanda, M. Prato, G. Almeida, I. Infante, L. Manna, *ACS Energy Letters* **2019**, *4*, 819-824.

[3] A. De Backer, K. H. W. van den Bos, W. Van den Broek, J. Sijbers, S. Van Aert, *Ultramicroscopy* **2016**, *171*, 104-116.
